# Multimodal Web-Based Telerehabilitation for Patients With Post–COVID-19 Condition: Protocol for a Randomized Controlled Trial

**DOI:** 10.2196/65044

**Published:** 2025-05-21

**Authors:** Aleksandar Tomaskovic, Vincent Weber, David T Ochmann, Elmo Wanja Neuberger, Ella Lachtermann, Alexandra Brahmer, Nils Haller, Barlo Hillen, Kira Enders, Viktoria Eggert, Peter Zeier, Klaus Lieb, Perikles Simon

**Affiliations:** 1 Department of Sports Medicine, Prevention and Rehabilitation Institute of Sport Science Johannes Gutenberg University Mainz Mainz Germany; 2 Department of Sport and Exercise Science University of Salzburg Salzburg Austria; 3 Division of Exercise and Movement Science Institute for Sport Science University of Göttingen Göttingen Germany; 4 Department of Clinical Psychology and Neuropsychology Institute of Psychology Johannes Gutenberg University Mainz Mainz Germany; 5 Department of Psychiatry and Psychotherapy Leibniz Institute for Resilience Research University Medical Center of the Johannes Gutenberg University Mainz Mainz Germany

**Keywords:** myalgic encephalomyelitis, chronic fatigue syndrome, ME/CFS, postexertional malaise, PEM, exercise intolerance, return to work, physiotherapy

## Abstract

**Background:**

Patients with post–COVID-19 condition (PCC) experience persistent, long-term health consequences following SARS-CoV-2 infection, including fatigue, hyperventilation, cognitive impairment, and limitations in daily activities. There is emerging evidence suggesting that exercise and respiratory therapy–based telerehabilitation is safe and could potentially improve physical capacity while reducing health care costs.

**Objective:**

This study aims to evaluate the superiority of a multimodal, symptom-titrated telerehabilitation program over standard care in patients with PCC who are severely affected, using the highest oxygen uptake rate (VO_2peak_ [mL/min/kg]) achieved during the cardiopulmonary exercise test (CPET) and minute ventilation/carbon dioxide production slope (VE/VCO_2_ [full slope]) as primary outcomes. In addition, this study seeks to provide novel insights into the clinical and physiological adaptations associated with PCC, informing future rehabilitation strategies.

**Methods:**

This prospective, randomized, waitlist-controlled trial was approved by the Rhineland-Palatinate Medical Association ethics committee. All procedures comply with the Declaration of Helsinki. This study comprises 3 examination time points, which include patient-reported outcomes, clinical assessments, and a CPET. It is structured into an 8-week intervention phase followed by an 8-week follow-up phase. Following baseline assessment, patients will be randomly assigned to either the intervention group (IG) or the control group (CG). During the intervention phase, IG participants will receive a web-based, multimodal, symptom-titrated telerehabilitation program consisting of sports medicine consultations, weekly teleconsultations, a structured pacing approach, and exercise and respiratory therapy. In contrast, CG participants will receive treatment as usual, which includes a single sports medicine consultation on healthy habits and a self-directed pacing approach for managing symptoms and daily activities. During the follow-up phase, IG participants will continue training independently without teleconsultations, whereas CG participants will undergo the same telerehabilitation intervention as the IG. A follow-up assessment will be conducted for both groups to evaluate long-term effects. This study adheres to the SPIRIT (Standard Protocol Items: Recommendations for Interventional Trials) guidelines and follows the Consensus on Exercise Reporting Template.

**Results:**

Recruitment began in August 2023 and was extended until March 2025. As of March 2025, 80 participants have been recruited, and data analysis is ongoing. Final results are expected by December 2025, with a cross-sectional analysis of baseline data anticipated by July 2025.

**Conclusions:**

This study is the first randomized controlled trial investigating the effectiveness of multimodal and symptom-titrated telerehabilitation in patients with PCC who are severely affected. The integration of various objective diagnostic systems will provide valuable insights into emerging postviral fatigue syndromes, supporting the development of CPET-based diagnostics, personalized rehabilitation strategies, and future research on long-term telerehabilitation effectiveness. The findings will be disseminated through peer-reviewed publications, professional networks, and patient advocacy groups to ensure scientific, clinical, and public impact.

**Trial Registration:**

German Clinical Trials Register (DRKS) DRKS00032394; https://drks.de/search/de/trial/DRKS00032394

**International Registered Report Identifier (IRRID):**

DERR1-10.2196/65044

## Introduction

### Background

Many individuals with COVID-19 continue to experience symptoms well beyond the acute phase of the disease, which can last for months or even years [1,2]. This persistent condition, known as post–COVID-19 condition (PCC), affects an estimated 6% to 15% of patients with COVID-19, and conservative estimates suggest that approximately 65 to 75 million people worldwide are experiencing PCC [3,4]. Consequences go beyond individual health, impacting social systems and the economy. For instance, the financial strain on the German health care and pension system was projected to reach €1.7 billion (US $1.96 billion) in 2021, with additional production losses and gross value-added losses amounting to €3.4 billion (US $3.91 billion) and €5.7 billion (US $6.56 billion), respectively [5].

PCC manifests as a multisystemic disorder with diverse clinical symptoms and can be categorized into 3 clusters: physical and cognitive impairments and mental disorders [3,6]. The most common symptoms among all the clusters are fatigue, hyperventilation, limitations in daily activities, cognitive impairments, and mental disorders [7]. Overall, 8% to 16% of patients with PCC meet the diagnostic criteria for myalgic encephalomyelitis or chronic fatigue syndrome (ME/CFS) because they experience both chronic fatigue and postexertional malaise (PEM) [8-10]. Chronic fatigue, defined by fatigue persisting for at least 6 months, and PEM, characterized by physical, mental, or emotional stress intolerance with exacerbation of symptoms even during everyday activities, are the leading symptoms of ME/CFS [11]. In addition to physical, mental, or emotional stress, PEM in patients with PCC is often triggered by hypersensitivity to food, chemicals, noise, light, extreme temperatures, inadequate sleep, or illness [12].

Regardless of the severity of acute COVID-19, the symptoms listed can persist for months and lead to an impaired quality of life and ability to work [7,13]. Female sex, older age, overweight status, smoking habits, preexisting comorbidities, and previous hospitalization or intensive care unit admission are significantly associated with a higher risk of developing PCC [14]. The etiology of PCC is believed to involve a combination of organ damage caused by the pathogen; a misdirected immune response; and additional mechanisms such as viral persistence, autoimmunity, endothelial dysfunction, and dysautonomia [15]. However, the specific physiological and biochemical pathomechanisms remain elusive [15,16].

Consequently, the focus of PCC treatment is on symptom management via multidisciplinary therapeutic approaches [17,18]. A survey in German rehabilitation centers for PCC showed the effectiveness of such measures in alleviating symptoms and improving health-related quality of life and work capacity [19]. Notably, 96 (72%) of 134 rehabilitation facilities reported longer durations of follow-up care for individuals with PCC than for those with other diseases. The most important therapeutic areas include exercise and respiratory therapy, psychotherapy, and therapeutic relaxation. A recent review discussed the positive physiological effects of endurance and strength exercise programs, such as immunomodulatory optimization and anti-inflammatory effects, which could reduce the long-term and health-related effects of PCC [20]. Several systematic reviews have examined the effects of exercise and respiratory therapy, particularly on physical capacity, hyperventilation, and quality of life, in patients with PCC [21-24].

Hyperventilation has been identified as a potential contributor to dyspnea in patients with COVID-19 and ME/CFS [25] as it may result in reduced carbon dioxide levels, leading to respiratory alkalosis and related symptoms such as breathlessness, dizziness, and fatigue. This mechanism is particularly relevant for patients with PCC experiencing persistent dyspnea as dysfunctional breathing patterns, including hyperventilation, may exacerbate respiratory symptoms and negatively impact overall quality of life [26]. Consequently, addressing hyperventilation through targeted respiratory therapy could be an important approach to improving hyperventilation and enhancing rehabilitation outcomes. In general, exercise and respiratory therapy have been shown to be safe and effective; however, physical activity or structured exercise may also trigger PEM in some patients [12].

Understanding PEM is important, especially for safe exercise therapy, and requires an analysis of the responses triggered by exercise. Objective pathological changes at the tissue level, particularly in blood and muscle tissue, can be observed following intense physical exertion in patients with PCC [27]. The cardiopulmonary exercise test (CPET), an objective assessment of cardiorespiratory fitness (CRF), oxygen uptake (VO_2_), and ventilatory efficiency during exercise, is used to diagnose dysfunction at the pulmonary, cardiac, and muscle level [28]. Patients with PCC who are severely affected have decreased levels of CRF, lower VO_2_ and ventilatory efficiency, and an impaired metabolic response during exercise and recovery [29-31]. Thus, the CPET serves as a valuable tool for the physiological characterization of patients with PCC, facilitating the prescription of personalized, symptom-titrated exercise programs.

Promising strategies for handling PEM in daily activities include pacing and the symptom-titrated approach to exercise therapy [32-34]. Both strategies focus on patient-centered care. Pacing therapy primarily aims to prevent the exacerbation of symptoms, especially fatigue and the onset of PEM, while promoting optimal activity levels for patients in their daily lives, whereas exercise therapy aims to improve their physical capacity. While pacing is widely used in the therapeutic treatment of patients with PCC and involves treatment as usual with a healthy lifestyle (eg, diet and sleep behavior) and drug treatment for accompanying symptoms, exercise therapy is controversially discussed, especially in patients with PCC who are severely affected, and is only used with reservations in standard care [35].

Given the widespread prevalence of PCC and its substantial impact on the health care system, telerehabilitation has emerged as a potentially cost-effective solution [36] for accessing a high number of patients irrespective of location and time [37,38]. Current systematic reviews on exercise and respiratory therapy–based telerehabilitation in patients with PCC [39,40] have reported improvements in quality of life and hyperventilation, as well as an increase in physical capacity.

### Objectives

Therefore, we developed a multimodal, personalized, and symptom-titrated telerehabilitation program that includes weekly teleconsultations, a pacing approach, and exercise and respiratory therapy for patients with PCC who are severely affected and unable to work. The primary aim of this study is to investigate the superiority of our telerehabilitation program compared to treatment as usual in terms of CRF (highest VO_2_ rate achieved during the CPET [VO_2peak_; mL/min/kg]) and hyperventilation (minute ventilation [VE]/carbon dioxide production [VCO_2_] slope [full slope]). In addition, the secondary aim is to explore the clinical and exercise physiology processes associated with PCC. A better understanding of postviral fatigue conditions can improve not only medical treatment but also the management of return to work for patients with PCC and ME/CFS.

## Methods

This study was prospectively registered in the German Clinical Trials Register on July 28, 2023 (registration: DRKS00032394).

### Ethical Considerations

This study was reviewed and approved by the ethics committee of the Rhineland-Palatinate Medical Association at the University Medical Center Mainz (registration: 2023-17082) in June 2023. The research was conducted in full compliance with institutional and ethical standards of the Declaration of Helsinki for research with human participants. All participants provided written informed consent before participation in the study. Participants were informed about the study purpose, procedures, potential risks and benefits, the voluntary nature of their participation, and their right to withdraw from the study at any time without consequences. Regarding any secondary analyses using existing data, the original consent and ethics approval explicitly covered the possibility of secondary analysis without requiring additional consent from participants. All collected data were fully anonymized before analysis. No personally identifiable information was stored or used in any publications or supplementary materials. Appropriate technical and organizational measures were implemented to protect participants’ confidentiality and data security. Participants did not receive any monetary compensation for taking part in this study. Participation was entirely voluntary without financial incentive. No identifiable images of study participants are included in the manuscript or supplementary materials. 

### Study Design

#### Overview

**Figure 1:**
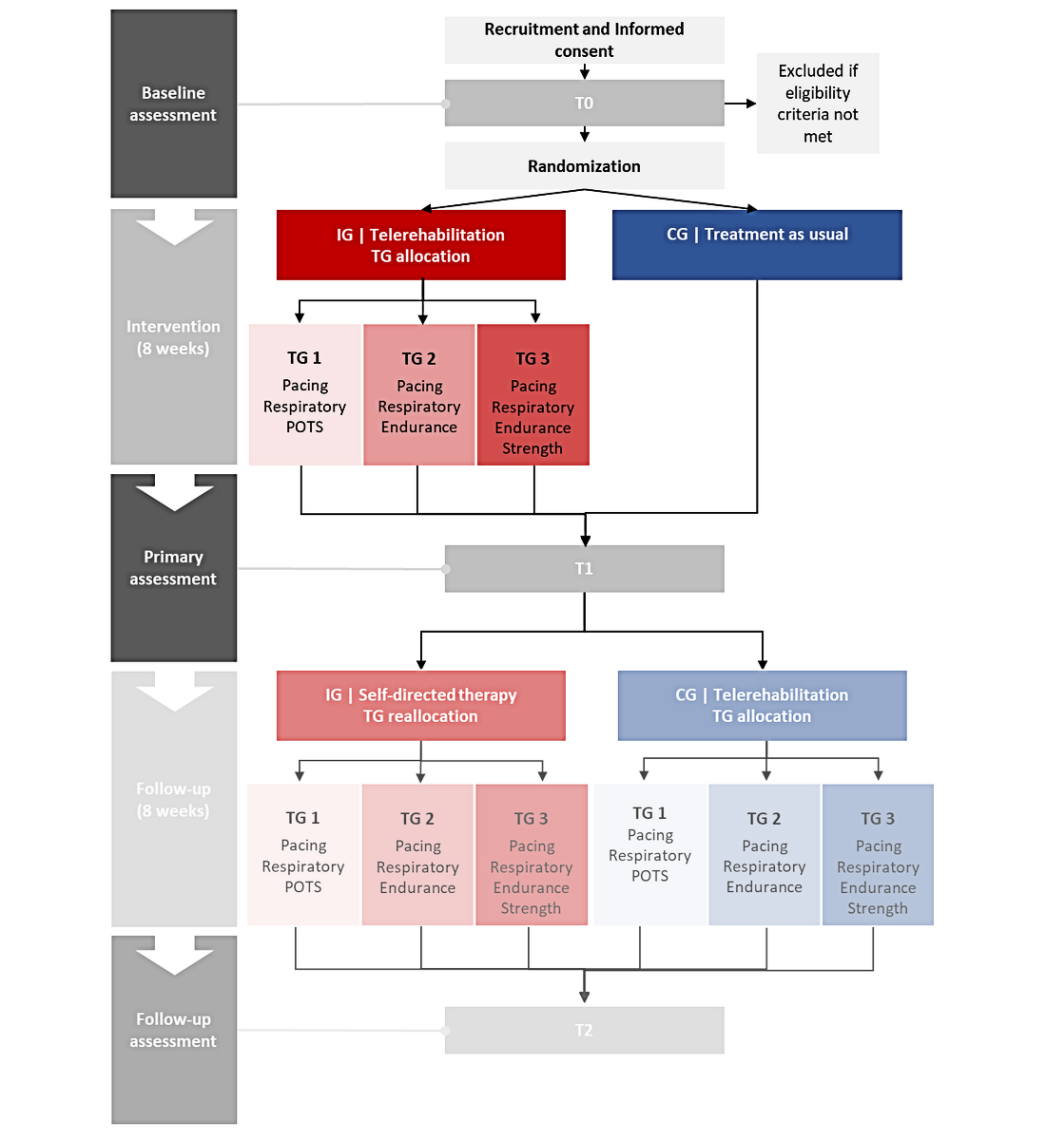
Study flowchart. The study consists of 3 assessment time points (T0-T2), including an 8-week intervention and an 8-week follow-up phase. Participants were allocated or reallocated to 3 training groups (TGs) based on baseline cardiorepiratory fitness parameters. The intervention included telerehabilitation (teleconsultation and web-based training), while the control group received usual care. CG: control group; Endurance: aerobic interval training; IG: intervention group; Pacing: pacing strategy for self-management of energy; POTS: postural orthostatic tachycardia syndrome training; Respiratory: respiratory training; Strength: strength training.

This study is designed as a single-center prospective randomized controlled trial (RCT) with a waitlist control design (Figure 1) and is conducted by the Department of Sports Medicine, Prevention, and Rehabilitation at Johannes Gutenberg University Mainz in cooperation with the German social accident insurance provider for nongovernmental health and social care institutions (German: *Berufsgenossenschaft für Gesundheitsdienst und Wohlfahrtspflege* [BGW]). The total study duration is 16 weeks and consists of an 8-week intervention phase and an 8-week follow-up phase with 3 examination time points: baseline assessment (T0) before the intervention, primary assessment (T1) after the intervention, and follow-up assessment (T2) after the follow-up phase. Participants will be informed about the objectives and risks of the study 1 week before T0 in written form (information document and informed consent form via email) and verbally at T0. They are required to provide their written consent before the baseline examination and, therefore, are not blinded to the study intervention. Patients will then undergo a comprehensive baseline assessment at T0 (questionnaires, clinical assessment, CPET, and muscle strength testing). A summary of the study phases, examination time points, and assessments is provided in Table 1. After the baseline assessment, participants are stratified by sex, age, and peak power output (PO; W/kg of body weight) during the CPET and then randomly assigned to either the intervention group (IG) or the control group (CG) by a researcher (BH) using a self-written R script (R Foundation for Statistical Computing; Multimedia Appendix 1).

The CPET is used to allocate the participants from the IG to 1 of 3 training groups (TGs; TG 1-TG 3) based on the allocation criteria (Table 2). After the intervention phase, participants in both groups (IG and CG) will undergo the same comprehensive examination at T1 as at T0 to evaluate the effects of the intervention on the IG and assign CG participants to the intervention and allocate them to 1 of the 3 TGs. In the follow-up phase, the CG participants undergo the same intervention as the IG during the intervention phase, whereas the IG (reallocated if necessary) continues the exercise and respiratory therapy in a self-directed manner. To evaluate the long-term effects of the intervention on the IG and the short-term effects on the CG, both groups will undergo the same comprehensive examination at T2.

**Table 1:** Overview of the study phases and groups, examination time points, and assessments.

			T0^a^		Intervention phase^b^				T1^c^		Follow-up phase^b^				T2^d^
					IG^e^ (T0-T1)		CG^f^ (T0-T1)				IG (T1-T2)		CG (T1-T2)		
**Enrollment**															
	Informed consent	✓													
	Eligibility criteria	✓													
	Randomization (stratified)	✓													
	Training group allocation	✓						✓							
**Questionnaires**															
	Nijmegen questionnaire	✓						✓						✓	
	DSQ-SF-PEM^g^ questionnaire	✓						✓						✓	
	Canadian Consensus Criteria	✓												✓	
	Bell scale	✓												✓	
	Brief Symptom Inventory–18	✓						✓						✓	
	Insomnia Severity Index	✓						✓						✓	
	Perceived Social Support Questionnaire	✓						✓						✓	
	Optimism-Pessimism Short Scale–2	✓						✓						✓	
	General Self-Efficacy Scale	✓						✓						✓	
	Satisfaction evaluation													✓	
**Clinical assessments**															
	Anamnesis^h^	✓													
	Anthropometric data^i^	✓						✓						✓	
	ECG^j^	✓						✓						✓	
	Blood pressure	✓						✓						✓	
	Pulmonary function test	✓						✓						✓	
	Clinical examination^h^	✓													
	Blood biomarkers^k^	✓						✓						✓	
	Hyperspectral imaging^l^													✓	
	Blood gas analysis													✓	
**Cardiopulmonary exercise testing**															
	Spiroergometry	✓						✓						✓	
	Lactate and cfDNA^m^ diagnostics^n^	✓						✓						✓	
**Muscle strength testing**															
	Hand grip strength	✓						✓						✓	
**Intervention**															
	Telerehabilitation^o^			✓								✓			

^a^T0: baseline assessment.

^b^8 weeks.

^c^T1: primary assessment.

^d^T2: follow-up assessment.

^e^IG: intervention group.

^f^CG: control group.

^g^DSQ-SF-PEM: DePaul Symptom Questionnaire–Short Form for Postexertional Malaise.

^h^Conducted by a study physician.

^i^Weight, height, and body composition.

^j^ECG: electrocardiography.

^k^The complete list of blood biomarkers can be found in Multimedia Appendix 2.

^l^Optical peripheral microcirculatory analysis.

^m^cfDNA: cell-free DNA.

^n^Capillary lactate (mmol/L) concentration and venous cfDNA concentration (ng/mL).

^o^Weekly teleconsultations and web-based exercise and respiratory therapy.

**Table 2:** Criteria for training group (TG) allocation based on the algorithm for prognostic and diagnostic stratification criteria for patients with chronic lung and heart disease described in the study by Guazzi et al [28]^a^.

Group	VO_2peak_^b^ (mL/min/kg)	PPO^c^ (W/kg of body weight)	VE/VCO_2_^d^ slope (full slope)
TG 1	<16	<1	>45
TG 2	16-20	1-1.5	30-45
TG 3	>20	>1.5	<30

^a^If 2 out of 3 variables correspond to one of the TGs, the study participant is assigned to that group. If all 3 variables are aligned with different groups, the group allocation is discussed individually with the study physician.

^b^VO_2peak_: highest oxygen uptake rate achieved during the cardiopulmonary exercise test.

^c^PPO: peak power output.

^d^VE/VCO_2_: minute ventilation/carbon dioxide production.

#### Participants

A total of 80 participants will be recruited by the Department of Sports Medicine, Prevention, and Rehabilitation at Johannes Gutenberg University Mainz in cooperation with the BGW and selected according to the inclusion and exclusion criteria defined in Textbox 1 [41,42]. The study population will consist of employees in the German health care or social services sector who are unable to work full or part time due to PCC. All participants will have a documented SARS-CoV-2 infection acquired through their work and experience persistent PCC symptoms lasting >12 weeks [2], leading to substantial impairment in daily functioning and occupational disability.

**Inclusion and exclusion criteria.**

**Inclusion criteria**

- Age of ≥18 years
- Signature of the consent form
- Proven SARS-CoV-2 polymerase chain reaction test provided by medical personnel
- Post–COVID-19 condition (PCC) diagnosis by the family or company physician (main diagnosis+ *International Classification of Diseases* diagnosis code U09.9)
- Occupational disability (full or part time) due to PCC
- Insured by the German social accident insurance provider for nongovernmental health and social care institutions

**Exclusion criteria**

- No internet access or inability or unwillingness to participate in the study
- Lack of capacity to consent or doubts about capacity to consent
- Participation in another rehabilitation program based on exercise and respiratory therapy (this means that individual passive physical therapy [eg, massage or thermotherapy], psychotherapeutic treatments, and visits to the physician are permitted)
- Red flags [41] and absolute contraindications for exercise training and physical activity [42]
- Participation in another study
- Inability to undergo performance diagnostics on the cycle ergometer
- Other unspecified factors discouraging study participation

#### Outcomes

The primary objective of this study is to investigate the effects of the intervention on CRF and hyperventilation. Evaluation of changes from baseline to primary assessment with respect to the IG and CG will involve (1) changes in CRF (VO_2peak_ [mL/min/kg]) and (2) changes in hyperventilation (VE/VCO_2_ slope [full slope]).

The secondary objective is to investigate the effects of exercise on other physiological, clinical, and psychosocial variables. Evaluation of changes from baseline to primary assessment with respect to the IG and CG will involve (1) changes in PO at maximal exertion (peak PO [W/kg of body weight]) and at the first and second ventilatory threshold (PO [W/kg of body weight]; if the ventilatory thresholds cannot be determined, the lactate thresholds will be used alternatively); (2) changes in VO_2_ efficiency, measured using the VO_2_ efficiency slope (OUES [VO_2_/VElog]), oxygen pulse (O_2_ pulse [mL/beat]), and oxygen cost of work (ΔVO_2_/ΔWR [mL/min/W]); (3) changes in ventilatory efficiency, evaluated using the cardiorespiratory optimal point (COP [VE/VO_2_min]), ventilatory equivalent for carbon dioxide (ECO_2_) and for oxygen (EO_2_), end-tidal carbon dioxide tension (PETCO_2_ [mmHg]) and end-tidal oxygen (PETO_2_ [mmHg]) tension, and Nijmegen questionnaire score; (4) changes in handgrip strength (HGS; kg); (5) changes in blood biomarker levels, evaluated using venous cell-free DNA (cfDNA; ng/mL) and capillary lactate (mmol/L) concentrations; (6) adherence to the intervention protocol (percentage of the prescribed intervention days); (7) changes in clinical condition and functional disability (frequency and severity of PEM, measured using the rating scale for perceived symptom severity [RPSS; described in the *Monitoring of Clinical Condition and Control of Training Load* section], the score on the DePaul Symptom Questionnaire–Short Form for Postexertional Malaise, the Canadian Consensus Criteria for diagnosis of ME/CFS, and the Bell chronic fatigue and immune dysfunction syndrome disability scale); and (8) changes in selected psychosocial variables (somatization, depression, and anxiety [Brief Symptom Index–18]; severity of insomnia [Insomnia Severity Index]; social support [short form of the Perceived Social Support Questionnaire]; optimism and pessimism [Optimism-Pessimism Short Scale–2]; and self-efficacy [General Self-Efficacy Scale]).

It should be noted that, except for the VE/VCO_2_ slope (full slope), which is calculated from the start of load to maximum exertion, and cardiorespiratory optimal point, which is the minimum ventilatory equivalent for oxygen during the CPET, all spiroergometric parameters are measured at rest, at the ventilatory (lactate) thresholds, and at maximal effort.

### Outcome Measures

#### Questionnaires

Validated questionnaires are completed at each examination time point before the clinical assessment, and the CPET records various disease- and health-related aspects. The questionnaires are administered via the WordPress Gravity Forms Plugin (Rocketgenius Inc) and are accessible to participants from the day of consent until T0 or 7 days before for T1 and T2. To minimize response fatigue and bias, participants are explicitly instructed to distribute questionnaire completion over several days rather than completing them all at once, ensuring better energy management and more accurate responses. However, the completion period is limited to a maximum of 7 days to maintain the validity of the responses at each test time point. Any questionnaires not completed within this time frame are finalized on-site before the clinical assessment and CPET.

The Nijmegen questionnaire will be used to evaluate symptoms of breathing pattern disorders and hyperventilation syndrome [43]. The frequency, duration, and severity of PEM are determined using the DePaul Symptom Questionnaire–Short Form for Postexertional Malaise [44]. In addition, 2 already established questionnaires for ME/CFS will be used. These are the Canadian Consensus Criteria [45], which represent a definition scheme for the clinical diagnosis of ME/CFS, and the Bell chronic fatigue and immune dysfunction syndrome disability scale [46], which represents a determination of functional disability. Due to the strong similarity in symptoms between PCC and ME/CFS [12,47], both questionnaires are also suitable for patients with PCC. The Brief Symptom Index–18 will be used to quantify somatization, depression, and anxiety [48]. The Insomnia Severity Index will be used to quantify the severity of insomnia symptoms [49]. The General Self-Efficacy Scale will be used to assess general optimistic self-belief [50]. The short form of the Perceived Social Support Questionnaire will be used to assess social support [51]. The Optimism-Pessimism Short Scale–2 will be used to measure the psychological trait of optimism-pessimism [52]. To assess participant satisfaction with our multimodal telerehabilitation program, we created and applied a satisfaction questionnaire based on previous process evaluations of our last 4 clinical trials [53].

#### Clinical Assessment

All participants undergo a clinical assessment at all 3 examination time points before the CPET. The aim is to perform risk stratification, particularly to identify participants at high risk of PEM, and perform specific analyses to better understand PCC. Measurements of height, body composition (InBody 3.0; Biospace), blood pressure (digital sphygmomanometer; ERKA), 12-lead electrocardiography (CardioPart 12 PC; AMEDTEC Medizintechnik GmbH), and pulmonary function (Body Box 5500; Medisoft GmbH) will be conducted by medical assistants at rest. Anamnesis and clinical examinations will be conducted by a blinded study physician (EL [internist]) according to the inspection, percussion, palpation, auscultation, and functional check algorithm. The results of the clinical assessments will be discussed with the physician and sports scientist to decide whether the participant is physically able to perform the CPET or whether they will be excluded from the study. In addition, venous blood analysis (Multimedia Appendix 2), blood gas analysis (Stat Profile Prime Plus; Nova Biomedical GmbH), and optical peripheral microcirculatory analysis with hyperspectral imaging (TIVITA Tissue System; Diaspective Vision GmbH) will be conducted. For venous blood analysis, 4.9 mL of blood are collected from the antecubital vein into a K3-EDTA Monovette (Sarstedt) 5 minutes before the CPET. For blood gas analysis, 50 µL of capillary blood are collected from the earlobe 5 minutes before and immediately after the CPET.

#### CPET Component

##### Spiroergometry

**Figure 2:**
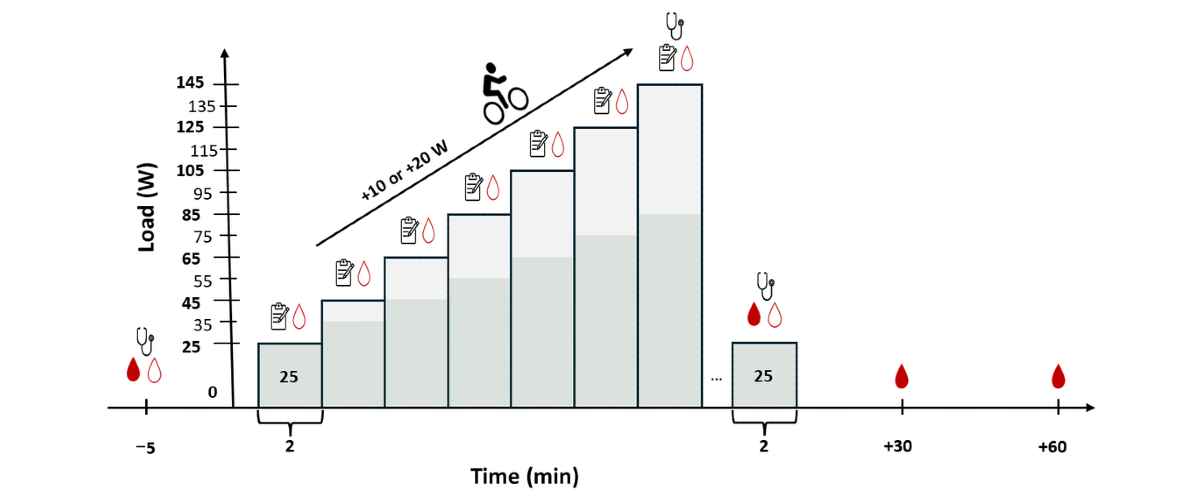
Illustration of the cardiopulmonary exercise test (CPET) protocols. The CPET protocol includes 2-minute stages starting at 25 W with load increments of 10 or 20 W per stage. Venous blood samples (filled drops), capillary blood samples (unfilled drops), blood pressure measurements, and rating of perceived exertion assessments are indicated.

To evaluate changes in CRF, hyperventilation, and other physiological exercise variables, spiroergometry will be conducted on an ER 900 PC cycling ergometer (Ergoline GmbH) using the Blue Cherry software (Geratherm Respiratory GmbH). The test protocols are designed to consist of 4 to 6 stages, each lasting 2 minutes, during which the exercise intensity (in W) is continuously increased at the end of each stage (Figure 2). In total, 2 test protocols with different exercise durations will be used. These protocols are tailored to the patient’s physical capacity and ensure a test duration of 8 to 12 minutes, thus maximizing the reliability of the VO_2peak_ determination [54,55]. All CPETs is carried out by a blinded sports scientist (DTO).

A 12-lead electrocardiogram is continuously monitored during the tests. VE, VO_2_, and VCO_2_ will also be continuously measured and analyzed using the breath-by-breath method. The rating of perceived exertion (RPE) will be assessed using the Borg scale (6-20) in the last 30 seconds of each exercise stage [56]. Testing is stopped when absolute or relative termination criteria (Textbox 2) are met. Primary termination criteria or subjective symptoms (eg, fatigue, leg fatigue, angina, and dyspnea) are documented immediately after completion of the CPET.

To provide accurate information on the data processing strategy to determine the VO_2peak_, we will follow the current guidelines for reporting of data processing strategies to determine maximum VO_2_ (Multimedia Appendix 3) [58]. The raw, unmodified data are filtered using a third-order Butterworth forward-backward low-pass filter with a 0.04-Hz cutoff implemented in the *spiro* package for R (version 0.0.4) [59] to determine the VO_2peak._ The VE/VCO_2_ slope (full slope) is calculated using linear regression analysis of VE and VCO_2_ with unmodified raw data throughout the load phase of the exercise test protocol (Multimedia Appendix 4) [55]. For the analysis of submaximal CRF and energy metabolism, the ventilatory thresholds [60] are defined based on established criteria (Multimedia Appendix 5).

**Absolute and relative termination criteria [54,57].**

**Absolute termination criteria**

- Decrease in systolic blood pressure of >10 mm Hg compared to the initial blood pressure despite an increase in stress with other signs of ischemia
- Definite angina pectoris
- Increasing cerebral symptoms (eg, ataxia, confusion, or presyncope)
- Signs of reduced peripheral perfusion (cyanosis or pallor)
- Technical reasons that make it impossible to adequately analyze the electrocardiogram or systolic blood pressure
- The study participant’s desire to terminate the exercise
- Persistent ventricular tachycardia
- ST segment elevation of at least 0.1 mV in leads without pathological Q waves (not augmented vector right or precordial lead V1)

**Relative termination criteria**

- Decrease in systolic blood pressure of >10 mm Hg compared to the initial blood pressure despite an increase in stress without other signs of ischemia
- Increasing angina pectoris
- Arrhythmias other than sustained ventricular tachycardia, including multifocal ventricular extrasystoles, triplets, supraventricular tachycardia, blockages, or atrial fibrillation
- Fatigue, breathlessness, wheezing, leg cramps, or claudication
- Development of a bundle branch block or intraventricular conduction delay that cannot be distinguished from ventricular tachycardia
- Reduced cadence of <40 revolutions per minute
- Arterial hypertension (250 mm Hg systolic or 115 mm Hg diastolic)
- ST segment or QRS complex changes, such as horizontal or descending ST segment depression (>0.2 mV) or pronounced change in position type

##### Lactate Diagnostics

For lactate diagnostics, capillary blood samples (20 μL) are taken from the earlobe 5 minutes before, during, and at the end of each exercise stage (last 30 seconds) and 3 minutes after completion of the CPET. Blood samples are measured immediately after the CPET through the polarographic determination method using an EKF Biosen S-Line measuring device (EKF Diagnostics GmbH) and analyzed using the winlactat 4.0 software (mesics GmbH). Lactate diagnostics include the measurement of the lactate concentration (mmol/L) and the determination of individual aerobic and anerobic thresholds for each participant using the *baseline +1.5 mmol/L method* according to Dickhuth et al [61].

##### cfDNA Diagnostics

At T0, venous blood samples are taken from the antecubital vein 5 minutes before, immediately after, 30 minutes after, and 60 minutes after the CPET using 4.9 mL of K3-EDTA Monovette (Sarstedt). In contrast to those at T0, the blood samples are only taken before and after the CPET at T1 and T2. The samples are centrifuged at 2500 × g for 15 minutes at room temperature. In total, 2 mL of plasma are taken and centrifuged a second time at 2500 × g for 15 minutes at room temperature. Plasma aliquots (150 and 550 µL) are collected in 1.5-mL tubes and stored at −20 °C according to established guidelines [62].

Concentrations of cfDNA will be measured using quantitative real-time polymerase chain reaction with unpurified plasma, as described previously [63]. The pipetting steps will be conducted using an automated pipetting system (ASSIST PLUS; INTEGRA Biosciences GmbH).

#### Muscle Strength Testing

HGS will be quantified during participants’ clinical assessments (before and after the CPET) by medical assistants using a Jamar hydraulic hand dynamometer (SH5001; Saehan Industries GmbH) following an established protocol by Nacul et al [64]. Participants are instructed to sit in an upright position and squeeze the handgrip as hard as possible, lasting 3 seconds, in 3 consecutive trials with 30 seconds of rest between trials. The entire procedure takes approximately 3 minutes to complete, including instructions. To examine changes in HGS over the course of the study, HGS_max_ is defined as the highest value of the 3 consecutive trials. In addition, we calculate the HGS_diff_ to determine the decrease in HGS between the 3 consecutive trials by subtracting the mean value between the second and third trials from the value of the first HGS trial, as described elsewhere [64].

### Intervention

#### Overview

Following the baseline examination (T0) and a 1-week washout period (recovery after the CPET), the IG will engage in therapist-led telerehabilitation for 8 weeks (intervention phase) followed by 8 weeks of self-directed therapy (follow-up phase). Concurrently, the CG will undergo treatment as usual for the initial 8 weeks, transitioning to the same therapist-led telerehabilitation as the IG in the follow-up phase. At each examination time point (T0-T2), after the clinical assessments and the CPET, all participants will undertake a sports medicine consultation with a sports scientist (DTO) to discuss the examination results, and they will be motivated toward a healthy lifestyle (eg, nutrition and stress control) and receive daily pacing recommendations. The results of the randomization (IG or CG) will be communicated to all participants within a few days after the baseline examination by a researcher (BH) via email.

#### Digital Infrastructure

**Figure 3:**
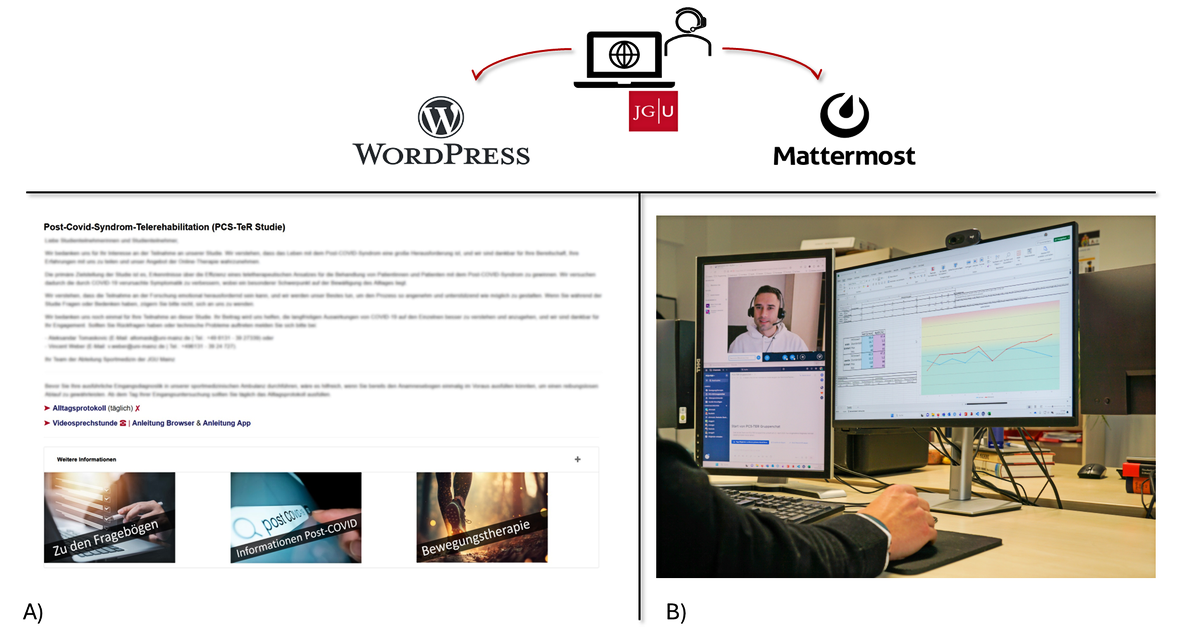
Digital infrastructure—(A) website for mobile and desktop use (WordPress; WordPress Foundation) and (B) mobile app and desktop application (Mattermost; Mattermost Inc). The author consents to the use of their image in this publication.

The digital infrastructure that will be used during the intervention phase is divided into 2 different parts. The first part is a central website designed for mobile and desktop use (WordPress; WordPress Foundation). The second part is the software Mattermost (mobile app and desktop application; Mattermost Inc), which is used for digital communication between therapists and patients (Figure 3).

The website consists of three main areas: (1) a diary for monitoring and controlling clinical condition and training load in addition to the questionnaires already described in the *Questionnaires* section; (2) an education area with short educational videos about PCC and exercise and respiratory therapy, including a detailed explanation and the pacing approach; and (3) a therapy program area including written materials (eg, weekly therapy plans, instructions for each type of training, and control of training intensity depending on the clinical condition).

All important information on the use of the website and Mattermost is provided in the study manual and communicated to the participants via email. In the intervention phase, the IG participants can use both the website and Mattermost. In contrast, CG participants can use only a restricted version of the website (without the education or therapy program areas) for daily reporting via the clinical condition and training load diary (CCTD).

#### IG Setup

On the basis of the CPET results and the algorithm listed in Table 2, IG participants are assigned to 1 of 3 TGs (TGs 1-3). Before the start of the intervention, all participants will take part in 2 video-based introductory sessions in the first week to get to know their personal sports therapist, learn how to use the digital infrastructure, and understand the therapy program (eg, therapy plan and monitoring of clinical condition).

#### Therapy Program

**Figure 4:**
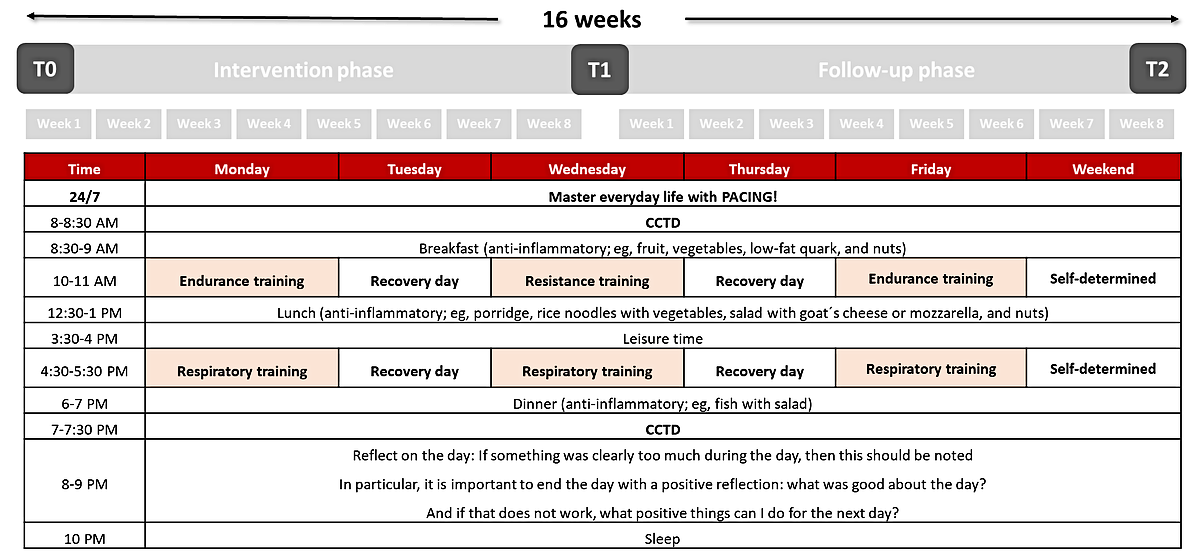
Exemplary weekly therapy plan for training group 3. The intervention and follow-up phases during the 16-week period are divided into 2 mesocycles (8 weeks) and 16 microcycles (1 week). The therapy plan consists of a combination of exercise and respiratory training types, training days, and recovery days. Participants are required to keep a daily clinical condition and training load diary (CCTD).

Due to the short duration of the intervention (8 weeks), and to achieve an optimal balance between the disease condition and the workload, a therapy plan (Figure 4) was created consisting of a combination of exercise and respiratory training types and training and recovery days and following the principle of periodization and cyclization. The intervention and follow-up phases during the 16-week period are divided into 2 mesocycles (8 weeks) and 16 microcycles (1 week). A change in TGs (eg, from TG 1 to TG 2) is planned for the end of the mesocycle if an objective improvement (or deterioration), measured through the CPET and evaluated using the described TG allocation algorithm (Table 2), occurs. Depending on the clinical conditions and the RPE during the training (eg, no worsening of clinical symptoms, and the subjectively perceived training intensity is too low), an individual group change within the mesocycle can also take place in consultation with the head physician (PS). The therapy plan can be individually adapted after each microcycle in teleconsultations with the sports therapist.

The cyclical alternation (Figure 4) between training and recovery days aims to consider the workload from the previous training session and provide the body with time to adapt. For example, if the intensity of the previous training session was too high, the study participants have time to reflect on this training stimulus and adapt, thus preventing the occurrence of PEM or not increasing its severity in a new training session. The training days include both exercise and respiratory training sessions held in the morning and afternoon. Because of the low exercise intensity, only TG 1 participants engage in daily training involving respiratory and postural orthostatic tachycardia syndrome (POTS) training, one in the morning and the other in the afternoon. The training sessions are conducted asynchronously for all participants (ie, without real-time therapist supervision).

The 3 TGs (Figure 1) follow an individual therapy plan. For TG 1 participants, the therapy plan includes respiratory and POTS training every day for at least 5 times a week. Respiratory training is based on the apnea training method [65] for increasing hypoxemia and ischemia tolerance. One respiratory training session consists of 3 sets of 4 repetitions, with a 2- to 3-minute pause between sets. One repetition consists of a deep inspiration maneuver followed by 4 maximum expirations and 4 submaximal inspirations to the level of resting inspiration. A POTS training session involves position change exercises for the arms, legs, and total body (eg, an exercise for standing up). The structure of each exercise consists of 2 to 3 sets with 5 to 10 repetitions, with a 1-minute pause between sets and 2 to 3 minutes between each exercise. For TG 2 and TG 3 participants, the therapy plan includes exercise training every other day but at least 3 times a week. TG 2 engages in endurance training as a substitute for POTS training. Moreover, TG3 performs both endurance and strength training instead of POTS training. Endurance training, designed as aerobic interval training and patient-centered training (eg, walking and cycling), entails 1 to 2 sets for TG 2 and 2 to 3 sets for TG 3, with a set pause of 2 to 3 minutes. Each set involves 8 to 10 repetitions with a work-to-rest ratio of 30:30 seconds or 30:60 seconds, with intensity control based on the modified Borg scale (0-10) for the RPE. Work intervals aim for an intensity between 4 and 6 (less strenuous to moderately strenuous), whereas (active) rest intervals target an intensity between 1 and 3 (very light to light). The individual progression or regression of the training load (eg, number of sets, intensity, and active or passive rest) depends on the patient’s exercise tolerance and can be carried out in consultation with a personal sports therapist.

**Figure 5:**
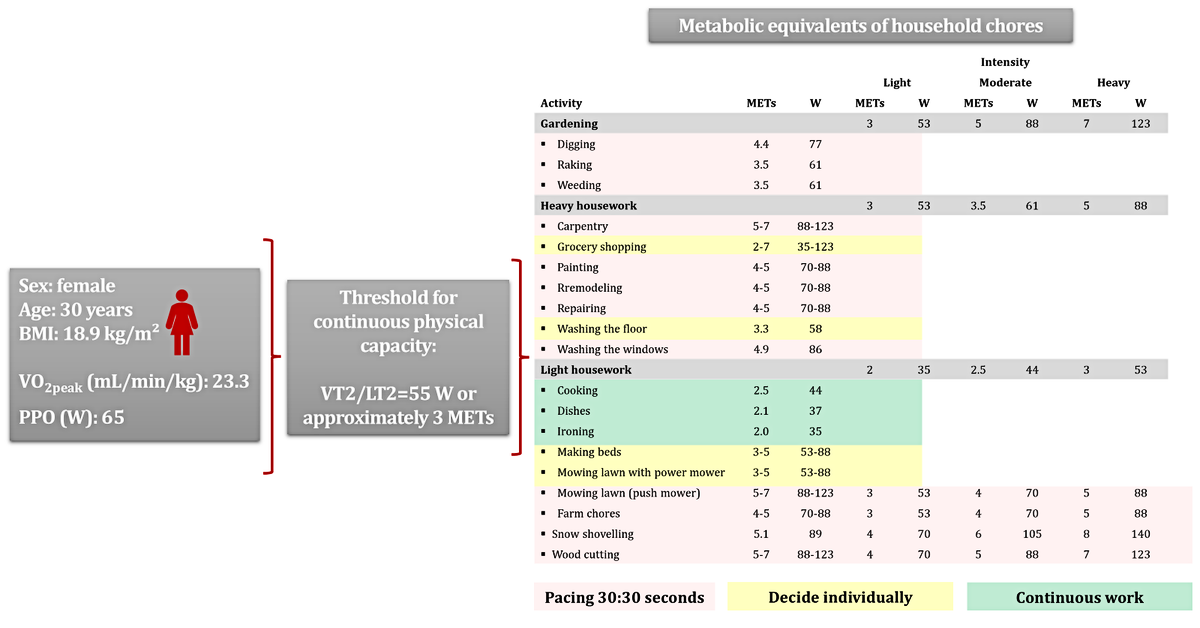
Objective pacing approach based on metabolic equivalent of task (MET) data from the work by Jetté et al developed by the Department of Sports Medicine, Prevention, and Rehabilitation at Johannes Gutenberg University Mainz. Objective pacing approach using cardiopulmonary exercise testing (CPET)–derived thresholds, illustrated with a 30-year-old female pilot participant. A continuous physical capacity threshold of 55 W (approximately 3 METs) corresponds to the individual’s ventilatory threshold 2/lactate threshold 2 (VT2/LT2). Household activities are categorized by intensity and color coded for pacing guidance (red: 30:30 seconds; yellow: individual pacing; green: continuous work). PPO: peak power output; VO_2peak_: highest oxygen uptake rate achieved during the cardiopulmonary exercise test.

All participants, both from the IG and CG, will be asked not only to practice additional pacing in their daily lives to be active but also to use their energy resources as efficiently as possible. To this end, based on the Royal College of Occupational Therapists pace, plan, and prioritize principle pacing approach [66] and the CPET, we developed a new objective pacing approach based on PO at the individual anaerobic threshold—ventilatory threshold 2/lactate threshold 2 (VT2/LT2)—as the threshold for continuous physical capacity and the metabolic equivalent of task (Figure 5 [67]) [67,68].

Our pacing strategy is based on the following six foundations:

1. Prevention of worsening of symptoms and occurrence of PEM: this requires the identification of extrinsic and intrinsic factors that trigger PEM, such as physical, cognitive, and emotional stressors.
2. Planning and prioritization of activities: this entails combinations of activity and rest phases and alternations among different activities (physical, cognitive, and emotional).
3. Pacing: all participants will receive a list of different physical activities sorted according to their metabolic equivalent of task.
4. Continuous physical activity is permitted only within the limits of dominant aerobic energy metabolism (<VT2/LT2) and must be subjectively well tolerated by the patient. If the desired physical activity is >VT2/LT2 in intensity, it should be performed only using the 30:30-second interval method (work-to-rest ratio of 1:1).
5. A cautious and gradual increase in activity can only occur if the clinical condition is good (refer to the *Monitoring of Clinical Condition and Control of Training Load section*).
6. The duration of work intervals and the work-to-rest ratio can be individually adjusted in consultation with a personal sports therapist depending on the patient’s clinical condition, exercise tolerance, and regeneration capacity.

During the intervention phase, all participants will have weekly teleconsultations (30-minute video calls) via Mattermost with their personal sports therapist. The teleconsultation is designed as a motivational and problem-solving interview with the following structure: (1) review of the previous week, highlighting all the positive aspects; (2) discussion of the current clinical and training-related form; and (3) outlook or intervention modifications for the upcoming week. In addition to the clinical condition (clinical symptoms) and training-related variables, the participants are asked to record any problems that arise during training or throughout the day. During the intervention phase, participants have the opportunity to use a chat function to consult with their personalized sports therapist for immediate support. In addition to weekly teleconsultations between therapists and participants, the therapy team should meet once a week to discuss the progress of therapy and current challenges, among other things. The therapy team consists of 5 therapists and a head physician (PS), and they are not blinded.

#### Monitoring of Clinical Condition and Control of Training Load

The daily recording of the clinical condition and the interactive adjustment of the exercise therapy aim to reduce or stabilize symptoms and adjust the training load to achieve the best training effects.

**Figure 6:**
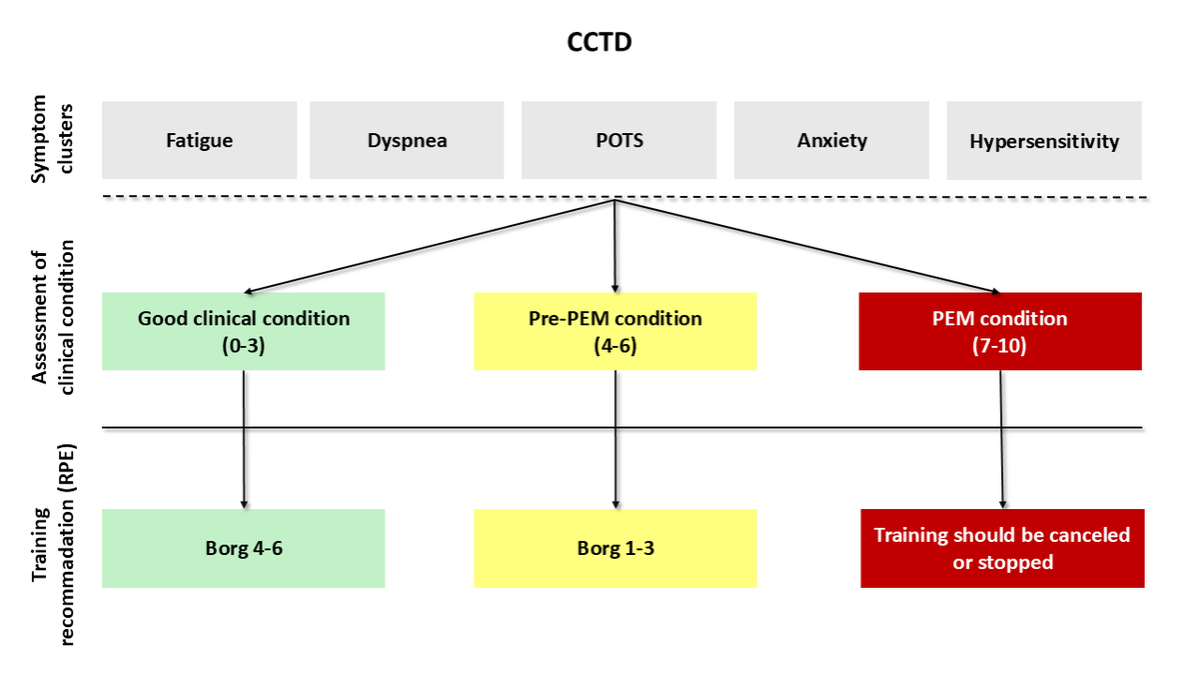
Clinical condition is assessed based on symptom severity (rating scale for perceived symptom severity), and training recommendations (rating of perceived exertion [RPE]) are derived accordingly. If any symptom cluster indicates a more severe condition (eg, pre–postexertional malaise [PEM] or PEM condition), the corresponding Borg value is applied. CCTD: clinical condition and training load diary; POTS: postural orthostatic tachycardia syndrome.

The training load is regulated based on clinical condition using a clear algorithm (Figure 6), which includes the assessment of the clinical condition based on the RPSS and the recommendation of the training intensity based on the RPE. The RPSS was developed through our clinical experience with PCC at the sports medicine outpatient clinic, Mainz [69], and through insights from international literature on symptom occurrence in PCC [7,12]. The successful application of the RPSS in practice constitutes its inaugural use in a scientific context. Both the RPSS and RPE will be documented in the CCTD. The RPSS comprises the following items: fatigue (influenza-like symptoms or general fatigue), dyspnea (breathing difficulties, headache, visual disturbance, or chest pain), POTS (high pulse, inability to stand, and need to lie down), anxiety (anxiety, panic, or avoidance), and hypersensitivity (to noise, light, temperature, touch, and muscle or nerve pain). Each symptom group is accompanied by a free-text field for individual comments that is rated on a scale from 0 to 10 (0=no symptoms; 1-3=mild symptoms; 4-6=moderate symptoms; 7-10=severe to maximum symptoms).

Participants are asked to complete the RPSS every day in the morning to monitor their clinical condition. Every day starting with even 1 symptom cluster between 7 and 10 (PEM condition) is counted as a PEM episode. On training days, the RPSS should be completed by the start of training at the latest. To avoid exercise-induced overload and exacerbation of clinical symptoms, patients should only exercise when each symptom cluster has a value of ≤6 (Figure 6). Ideally, each symptom cluster should have a value between 0 and 3 (good clinical condition); then, patients can train with an RPE of 4 to 6 to maintain good clinical condition and provide an effective training stimulus. Even if only 1 symptom cluster has a value between 4 and 6 (pre-PEM condition), the RPE value must be set to 1 to 3 during training. However, if any of the RPSS symptom clusters have a value between 7 and 10 (PEM condition), the upcoming exercise training session should be canceled. In addition, if symptoms increase by ≥7 values during training, the current training session should be stopped. Finally, an increase in symptoms of ≥2 values in more than one symptom cluster should also be considered for training load management. The possible cause for this increase should be investigated via teleconsultation. After the training session or at the end of the day at the latest, participants are required to document their training (type; duration; intensity; and, if applicable, commentary) in the CCTD.

#### CG Setup

All the questionnaires, clinical assessments, and CPET scores at each examination time point correspond to the IG. After T0, the CG participants receive an email with written information explaining the importance of self-management of symptoms and activities through the pacing approach. CG participants will be motivated toward a healthy lifestyle (eg, nutrition and stress control) and receive pacing recommendations but will not receive web-based exercise or respiratory therapy or teleconsultations. The term *treatment as usual* refers to clinical assessment, the CPET, a sports medicine consultation on healthy habits, and the pacing approach. After T1, the CG will undergo the same intervention as the IG but in the follow-up phase.

### Data Management

VW and AT are responsible for data management and analysis and are blinded. All analogue, digital, and biological study materials and data are pseudonymized. All the data entries and sensitive information about the participants are password protected and accessible only to the assigned researchers (VW and AT).

### Statistical Analysis Plan

For data storage, transformation, and analysis, Microsoft Excel (version 2401; Microsoft Corp) and R (version 4.4.0) will be used. VW and AT do not know which IG receives which intervention (TG 1, TG 2, or TG 3). Standard statistical procedures will be performed according to the intention-to-treat principle.

Descriptive statistics will be used for all outcome variables at each test time point to obtain an overview of the changes in all relevant data. Descriptive statistics will include absolute and relative frequencies for categorically scaled data and means, 95% CIs, and SD or medians, 95% CIs, and IQRs for continuously scaled data. The normality of the distribution will be tested using the Shapiro-Wilk test and visual inspection of the data. The homogeneity of variance will be tested using the Levene test. In the case of a nonnormal distribution, the data will be log-transformed before statistical analysis to assess normality and variance homogeneity; alternatively, more robust statistical models such as mixed-effects models will be used.

A repeated-measure ANOVA or a linear mixed-effects model will be used to determine the differences between all groups and time points [70,71]. The primary analysis will focus on the comparison between the IG and CG at T0 and T1 to evaluate the superiority of telerehabilitation over usual care while minimizing potential waiting bias. Participants who transition from the CG to the IG (waiting group) will be analyzed separately to assess whether delayed intervention yields similar effects. In addition, the IG will be analyzed during the follow-up phase as these participants will continue training without therapeutic support, allowing for an exploration of long-term intervention sustainability.

The statistical significance will be set at α<.05. The magnitude of the changes between time points, as well as the magnitude of the differences in changes between the groups, will be assessed using effect sizes. The threshold values for effect sizes will be 0.2 (small), 0.6 (moderate), 1.2 (large), and 2.0 (very large) [72,73]. In addition, 95% CIs for the between-group differences in changes will be estimated, and magnitude-based inferences will be made with reference to the smallest worthwhile change, which is calculated as 0.2 multiplied by the between-subject variation at T0 [73]. Correlations between variables will be assessed using parametric (Pearson) and nonparametric (Spearman rank) tests depending on the distribution of the variables. In the case of multiple comparisons, *P* values will be adjusted using appropriate procedures.

The sample size estimation is based on an a priori power analysis (repeated measures; interaction between group [intervention vs control] and time [before vs after the intervention]) performed using G*Power (version 3.1.9.7). Assuming a power of 0.8 and a medium to large effect (Cohen *f*=0.35) on the VO_2peak_ (in accordance with the work by Bacon et al [74]), 67 participants are needed. Considering the calculated required sample size and a dropout rate of 15% to 20% [53,75], the target number of study participants is set at a total of 80, or 40 participants per group.

Adherence to the intervention is defined as the percentage of training sessions completed of the prescribed training sessions [75]. Participants who drop out of the intervention or complete <60% (ie, <17 of the predefined 24 training sessions for TG 1) of the prescribed training are defined as dropouts. For those in the IG, adherence during the follow-up phase will also be tracked to assess sustained engagement without therapeutic supervision. Adherence to the training requirements will be assessed based on the information provided by the participants via the CCTD. Participants will continue to be monitored after their withdrawal from the study if they agree.

### Publication Strategy

The analysis strategy and publication plan include a comprehensive cross-sectional analysis of baseline assessments (T0) aimed at thoroughly characterizing participants’ clinical status (eg, symptoms, functional limitations, and comorbidities) and exercise physiology profiles (eg, CRF, ventilatory efficiency, and VO_2_ efficiency). This analysis will encompass clinical history related to COVID-19 (eg, severity of COVID-19, duration of hospitalization, and previous rehabilitation) along with data from patient-reported outcome measures, clinical assessments, and exercise physiological measurements. These baseline data will be reported separately in a dedicated observational cross-sectional paper, providing a detailed clinical and exercise physiological characterization of patients with PCC who are severely affected.

The RCT analysis will primarily compare outcomes between the IG and CG. In addition, subgroup analyses will be conducted to assess differences in therapeutic efficacy across key patient categories, including hospitalized versus nonhospitalized patients and patients with PCC with versus without ME/CFS. The inclusion of these subgroup analyses aims to enhance comparability with other PCC rehabilitation trials and provide deeper insights into factors influencing rehabilitation outcomes.

Furthermore, the collected data will be used for various analyses in basic research aimed at gaining insights into critical aspects such as the molecular characterization of PCC pathogenesis, the pathophysiology of PCC, and the pathomechanisms of PEM. These investigations will contribute to a more comprehensive understanding of PCC and support the development of more targeted and effective rehabilitation interventions.

This study adheres to the SPIRIT (Standard Protocol Items: Recommendations for Interventional Trials) guidelines [76] (Multimedia Appendix 6) and follows the Consensus on Exercise Reporting Template [77].

## Results

Recruitment for the study began in August 2023 and was initially planned for 12 months. On December 27, 2024, a protocol amendment was approved, extending the recruitment period until March 2025. Updated study information is available in the German Clinical Trials Register [78]. No further modifications to the registered study are planned, and no results have been published yet.

As of March 2025, 80 participants have been successfully recruited, and data analysis is ongoing. Final results of the RCT are expected to be published by winter 2025. A comprehensive cross-sectional analysis of the baseline assessments (T0) is expected to be published by summer 2025.

The study findings will be submitted to peer-reviewed journals and presented at scientific conferences, ensuring objective reporting regardless of the study outcome.

## Discussion

### Expected Findings

To our knowledge, the Mainz PCC telerehabilitation study is the first RCT to investigate the effects of a multimodal, personalized, symptom-titrated telerehabilitation program on CRF and hyperventilation in patients with PCC who are severely affected and have work disability. By integrating personalized teleconsultations, exercise therapy, and respiratory interventions, we expect improvements in VO_2peak_ (mL/min/kg) and the VE/VCO_2_ slope, along with better management of PCC symptoms such as chronic fatigue and PEM. While previous studies have shown that telerehabilitation can enhance quality of life and symptom management in patients with PCC, its application in individuals who are severely affected remains largely unexplored. Moreover, previous interventions have relied primarily on self-reported outcomes and have been often conducted in mildly to moderately affected patients with PCC. Our study bridges this gap by integrating objective outcome measures, including CPETs and biomarker analysis, to provide a comprehensive evaluation of telerehabilitation effectiveness in this specific cohort. In contrast to standard telerehabilitation approaches, our intervention dynamically adapts the training load to the subjectively perceived symptom intensity, ensuring a symptom-titrated approach tailored to the current clinical condition of each patient. In addition to the expected improvements in VO_2peak_ and the VE/VCO_2_ slope, this study will provide new insights into clinical and physiological adaptations associated with PCC and provide a structured framework for personalized telerehabilitation programs.

### Comparison to Prior Work

Exercise and respiratory therapy–based rehabilitation programs have been shown to improve physical capacity, hyperventilation, and health-related quality of life in patients with PCC. However, their effects on fatigue remain inconclusive [23,24]. A critical gap in previous research is the lack of evidence regarding the interaction between exercise training and fatigue, particularly in minimizing the risk of exercise-induced symptom exacerbation. While exercise and respiratory training are essential for improving CRF and ventilatory efficiency, pacing strategies have gained attention as a promising approach for managing PEM in daily activities [32].

Despite the growing body of research supporting exercise-based rehabilitation for PCC, previous interventions have largely relied on self-reported outcomes and have primarily been conducted in patients with mild to moderate disease severity [23,24]. Furthermore, most existing studies lack objective outcome measures, such as CPETs, lactate diagnostics, and muscle strength assessments, which are critical for accurately quantifying physiological responses and intervention efficacy [79-91]. To date, only 4 studies have incorporated objective outcome measures of CRF and clinical condition in patients with PCC [92-95]. While telerehabilitation has been shown to be a safe alternative to conventional rehabilitation [39,40], its overall efficacy remains uncertain. A recent meta-analysis of 16 RCTs found no significant improvements in clinical symptoms, pulmonary function, psychological well-being, or quality of life [96].

Building on these previous findings, our study introduces a novel, symptom-titrated telerehabilitation program that is designed to dynamically adjust the training load based on individual clinical responses. By integrating objective diagnostic tools, weekly teleconsultations, and structured pacing strategies, our approach seeks to address the shortcomings of previous telerehabilitation interventions and establish a personalized telerehabilitation program for patients with PCC who are severely affected.

### Strengths and Limitations

A key strength of this study is its comprehensive assessment strategy, which includes objective outcome measures rather than relying solely on self-reported outcomes. By incorporating CPETs and other objective physiological measures, we aim to quantify the physiological adaptations triggered by telerehabilitation and provide robust evidence on exercise tolerance in patients with PCC who are severely affected.

In addition, risk stratification and individualized exercise prescription are major methodological strengths. Our baseline assessment systematically classifies patients based on CRF, performance, and ventilatory efficiency, ensuring that telerehabilitation is personalized and adapted to each patient’s specific condition [29,33,97]. This contrasts with previous studies that applied generic exercise protocols [79-91], which may not be appropriate for individuals with fluctuating symptoms and severe functional limitations.

Compared to traditional face-to-face rehabilitation, telerehabilitation presents several advantages. First, it allows for remote delivery of high-quality rehabilitation by health care professionals, including physicians, physiotherapists, and sports scientists [36-38]. This is particularly valuable for patients with PCC who are severely affected, who may face mobility restrictions and difficulties accessing in-person rehabilitation programs. Second, telerehabilitation provides a scalable and cost-effective solution, addressing health care system limitations and the growing demand for PCC rehabilitation services.

Our previous research has demonstrated that exercise-based telerehabilitation is safe, with both short-term [53,75,98,99] and long-term benefits [100] in various patient populations. Notably, no serious adverse events were reported, and the dropout rate remained relatively low (15%-20%). This evidence supports the feasibility and clinical applicability of a symptom-titrated telerehabilitation approach, particularly for patients with limited access to conventional rehabilitation.

Thus, our multimodal telerehabilitation program—which combines objective diagnostics, personalized symptom-titrated exercise therapy, and teleconsultations—represents a promising alternative to standard rehabilitation or a valuable adjunct to outpatient physiotherapy and self-directed therapy. By addressing critical gaps in prior work and implementing a robust methodological design, this study has the potential to advance telerehabilitation as a viable rehabilitation strategy for patients with PCC, particularly those with severe disease manifestations.

Despite the strengths of this study, certain limitations must be acknowledged. A key challenge in our approach is the introduction of asynchronous exercise training, where participants engage in rehabilitation without real-time supervision from therapists. Previous telerehabilitation interventions have predominantly relied on synchronous models, allowing for direct therapist interaction during exercise sessions [81,95,101]. While the asynchronous format enhances accessibility and flexibility, particularly for patients with PCC who are severely affected, it may also pose challenges in adherence and compliance as PCC symptoms can fluctuate daily. To mitigate this issue, we integrated weekly teleconsultations via Mattermost, providing participants with opportunities to seek guidance, discuss symptoms, and adjust their rehabilitation plans accordingly. However, the absence of real-time therapist feedback may still present risks, particularly in individuals considered vulnerable. Future studies should explore the potential integration of live internet-based supervision or automated biofeedback mechanisms to optimize adherence and safety. Another limitation of this study is the extensive number of questionnaires, which participants must complete at multiple test time points. Although we have implemented measures to minimize response fatigue—such as allowing participants to distribute questionnaire completion over several days—the risk of response depletion remains. In addition, an important limitation of this study is the reliance on self-reported adherence and symptom tracking through the CCTD. While the digital training diary and RPSS offer valuable insights into training loads and symptom fluctuations, they lack objective physiological monitoring, such as heart rate variability, oxygen saturation, or movement data. Without these objective markers, it is challenging to precisely quantify exercise intensity and patient responses. Previous research has highlighted the potential benefits of wearable technology, which could enhance exercise monitoring by providing real-time physiological feedback [102,103]. Although this study does not incorporate such technology, future research should explore the use of wearable sensors and mobile health apps to enable real-time tracking and personalized exercise adjustments, thereby improving intervention precision. However, using digital technologies such as the internet, PCs, smartphones, or smartwatches for monitoring clinical condition and training load control may present challenges [104], especially for populations considered vulnerable, such as patients with PCC. Finally, a further limitation is the potential confounding effects of concurrent medical treatments. Participants may receive additional interventions during the study period, including pharmacological therapy, relaxation treatments (eg, massage), or other symptom management strategies, which could influence outcomes. While we have minimized this risk by excluding individuals undergoing structured physiotherapy or other exercise-based interventions, this study cannot fully control for the impact of all external treatments. Future research should consider subgroup analyses to examine how concurrent treatments interact with telerehabilitation outcomes, enabling a more comprehensive understanding of multimodal rehabilitation strategies.

### Dissemination Plan

The findings of the Mainz PCC telerehabilitation study will be disseminated through three channels:

1. Scientific community: the results will be published in peer-reviewed journals (eg, JMIR) and presented at national (eg, the German Long COVID Symposium) or international conferences on rehabilitation and digital health.
2. Clinical and professional outreach: the findings will be integrated into clinical guidelines (eg, the German
  Long/Post-COVID—Living Guideline
  ), professional workshops, and webinars for health care providers (eg, BGW specialist meeting) in collaboration with rehabilitation specialists and patient organizations (eg, the German organization for patients with ME/CFS [*Bundesverband ME/CFS - Fatigatio*]).
3. Public and patient engagement: lay summaries, infographics, and media releases will be shared via our website, patient forums, and social media, ensuring accessibility for patients with PCC and ME/CFS.

### Conclusions

The Mainz PCC telerehabilitation study aims to provide insights into the clinical and exercise physiological effects of an 8-week multimodal, personalized, and symptom-titrated telerehabilitation program for patients with PCC who are severely affected. The effectiveness of the intervention on CRF and ventilatory efficiency will be primarily assessed through VO_2peak_ and the VE/VCO_2_ slope. Furthermore, this study aims to determine whether weekly teleconsultations can enhance treatment adherence compared to self-directed rehabilitation approaches.

While this study is not designed to establish causal relationships, its findings may support the further development of CPET-based diagnostic approaches, risk stratification models, and personalized exercise and respiratory therapy prescriptions. Moreover, the results may provide preliminary evidence for evaluating work ability and rehabilitation planning in patients with PCC and ME/CFS who are severely affected. Future research should build on these findings to validate the long-term effectiveness and safety of telerehabilitation and refine rehabilitation strategies for postviral fatigue conditions.

## Appendix

Multimedia Appendix 1 R script for the stratified randomization.

Multimedia Appendix 2 Venous blood parameters for the Mainz post–COVID-19 condition telerehabilitation study.

Multimedia Appendix 3 Reporting on data processing strategies for determining the highest oxygen uptake rate achieved during the cardiopulmonary exercise test.

Multimedia Appendix 4 Reporting on data processing strategies for determining the minute ventilation/carbon dioxide production slope (full slope).

Multimedia Appendix 5 Standard method (V-slope method) and auxiliary criteria for determining the first and second ventilatory thresholds.

Multimedia Appendix 6 SPIRIT (Standard Protocol Items: Recommendations for Interventional Trials) checklist.

## Abbreviations

BGW: Berufsgenossenschaft für Gesundheitsdienst und Wohlfahrtspflege (German social accident insurance provider for nongovernmental health and social care institutions)
CCTD: clinical condition and training load diary
cfDNA: cell-free DNA
CG: control group
CPET: cardiopulmonary exercise test
CRF: cardiorespiratory fitness
HGS: handgrip strength
IG: intervention group
ME/CFS: myalgic encephalomyelitis or chronic fatigue syndrome
PCC: post–COVID-19 condition
PEM: postexertional malaise
PO: power output
POTS: postural orthostatic tachycardia syndrome
RCT: randomized controlled trial
RPE: rating of perceived exertion
RPSS: rating scale for perceived symptom severity
SPIRIT: Standard Protocol Items: Recommendations for Interventional Trials
TG: training group
VCO_2_: carbon dioxide production
VE: minute ventilation
VO_2_: oxygen uptake
VO_2peak_: highest oxygen uptake rate achieved during the cardiopulmonary exercise test
VT2/LT2: ventilatory threshold 2/lactate threshold 2

## Data Availability

The datasets generated or analyzed during this study are not publicly available due to participant confidentiality and data protection regulations but are available from the corresponding author on reasonable medical or scientific request in anonymized form.
