# Supplementary material for: Multimodal Web-Based Telerehabilitation for Patients With Post–COVID-19 Condition: Protocol for a Randomized Controlled Trial
Source: JMIR Res Protoc. 2025 May 21;14:e65044. doi: 10.2196/65044 (PMC12138299; doi:10.2196/65044)
Supplement: Multimedia Appendix 1 [file resprot_v14i1e65044_app1.pdf]

Multimedia Appendix 1: R Script for the stratified randomization

INSTALL PACKAGES & LOAD LIBRARIES ——————————————————

Check if ‘pacman’ package is available, if not, install it

```
if (!require("pacman")) install.packages("pacman")

## Lade nötiges Paket: pacman
```

Load required libraries

```
pacman::p_load(tidyverse, readxl, here)
```

SET PATHS ——————————————————————————————

Change the working directory to the parent directory

```
here::i_am("StratifiedRandomization_PCSTER.R")

## here() starts at /Randomisierung

wpath = here::here()
```

LOAD DATA ——————————————————————————————

Read the previous assignments data and arrange by numeric part of Studien_ID

```
prev_assignments = read_csv2(paste(wpath,"/Randomisierung_cleaned.csv", sep = "")) %>%
 arrange(as.numeric(sub("PCSTER-", "", Studien_ID)))

## ℹ Using "','" as decimal and "'.'" as grouping mark. Use `read_delim()` for more control.

## Rows: 105 Columns: 7
## ── Column specification ────────────────────────────────────────
## Delimiter: ";"
## chr (7): Studien_ID, Ambulanz_ID, name, Geschlecht, age_strat, maxRelLast_st...
##
## ℹ Use `spec()` to retrieve the full column specification for this data.
## ℹ Specify the column types or set `show_col_types = FALSE` to quiet this message.
```

Load the XLSX file containing new patient data

```
xlsx_file = paste(dirname(wpath), "/Studien_Dokumente/PCSTeR_Probanden_Informationen.xlsx", sep = "")
tdf = readxl::read_xlsx(xlsx_file)
```

DATA PROCESSING ———————————————————————————

```
table_data <- prev_assignments %>%
 group_by(Group, Geschlecht, age_strat, maxRelLast_strat) %>%
 summarise(Count = n()) %>%
 spread(Group, Count, fill = 0)
```

```
## `summarise()` has grouped output by 'Group', 'Geschlecht', 'age_strat'. You can
## override using the `.groups` argument.
```

Create all possible combinations for Geschlecht, age_strat, and maxRelLast_strat

```
combinations <- expand.grid(
  Geschlecht = c("w", "m"),
  age_strat = c("old", "young"),
  maxRelLast_strat = c("high", "low")
)
```

Combine the dataframes using bind_rows and keep distinct rows

```
combined_data <- bind_rows(table_data, combinations) %>%
  distinct(Geschlecht, age_strat, maxRelLast_strat, .keep_all = TRUE)
```

Process the new patient data

Create dichotomous variables for age & maxRelLast based on median

```
tdf = tdf %>%
  select(Studien_ID, Ambulanz_ID, Alter, Geschlecht, RelMaxLast) %>%
  drop_na() %>%
  mutate(med_age = median(Alter)) %>%
  mutate(age_strat = case_when(Alter > med_age ~ "old",
                               Alter <= med_age ~ "young")) %>%
  mutate(maxRelLast_med = median(RelMaxLast)) %>%
  mutate(maxRelLast_strat = case_when(RelMaxLast > maxRelLast_med ~ "high",
                                      RelMaxLast <= maxRelLast_med ~ "low")) %>%
  select(Studien_ID, Ambulanz_ID, Geschlecht, age_strat, maxRelLast_strat) %>%
  arrange(as.numeric(sub("PCSTER-", "", Studien_ID)))
```

Select only new patients

```
new_patients = tdf %>%
  filter(!Ambulanz_ID %in% prev_assignments$Ambulanz_ID) %>%
  mutate(Group = NA_character_)
```

STRATIFIED RANDOMIZATION ———————————————————————

Function for stratified random sampling (checks the distribution in each strata)

```
random_assignment <- function(strata) {
  if (is.na(strata$control) || is.na(strata$intervention) || strata$control == strata$intervention) {
    return(sample(c("control", "intervention"), 1))
  } else {
    return(ifelse(strata$control < strata$intervention, "control", "intervention"))
  }
}
```

Loop through each of the new patients and assign them to groups

```
if(nrow(new_patients) != 0){
  for(i in 1:nrow(new_patients)){
    new_people_assigned <- new_patients[i,] %>%
      group_by(Geschlecht, age_strat, maxRelLast_strat) %>%
      mutate(Group = random_assignment(combined_data %>%
```

```
                    filter(Geschlecht == new_patients[i,]$Geschlecht,
                        age_strat == new_patients[i,]$age_strat,
                        maxRelLast_strat == new_patients[i,]$maxRelLast_strat))) %>%
   ungroup() %>%
   mutate(Ambulanz_ID = as.character(Ambulanz_ID))

  # add new patients to previously assign patients
  prev_assignments = bind_rows(prev_assignments, new_people_assigned)
 }
} else {
 print("No new patients!")
}
```

Summarize and spread strata distribution data with new assignments

```
## `summarise()` has grouped output by 'Group', 'Geschlecht', 'age_strat'. You can
## override using the `.groups` argument.
```

SAVING RESULTS TO CSV ——————————————————————–
