# Supplementary material for: Multimodal Web-Based Telerehabilitation for Patients With Post–COVID-19 Condition: Protocol for a Randomized Controlled Trial
Source: JMIR Res Protoc. 2025 May 21;14:e65044. doi: 10.2196/65044 (PMC12138299; doi:10.2196/65044)
Supplement: Multimedia Appendix 2 [file resprot_v14i1e65044_app2.pdf]

Multimedia Appendix 2: Venous blood parameters

SARS-CoV-2 Related Parameters

SARS-CoV-2 TrimericS IgG, SARS-CoV-2-Ak (nucleocapsid)

Hematologic Parameters

Leucocytes, erythrocytes, red cell distribution width (RDW), hemoglobin, hematocrit, MCV, MCH, MCHC, platelets, mean platelet volume (MPV), neutrophils, lymphocytes, monocytes, eosinophils, basophils, blasts, myelocytes, metamyelocytes, total protein

Electrolyte and Mineral Parameters

Sodium, potassium, calcium, magnesium.

Parameters of renal function

Serum creatinine, glomerular filtration rate (CKD-EPI), uric acid, urea, cystatin C (IFCC), glom. Filtration rate (from cystatin C) (CAPA)

Liver Function Parameters

GOT (ASA, AST), GPT (ALAT, ALT), gamma-GT

Cardiovascular Markers

NTproBNP, LDH troponin I (high sensitive), titin

Autoantibodies

β1 and β2 adrenergic receptors (AdR) AB, M3 and M4 acetylcholine receptors (AChR) AB, Endothelin A-Rez. AB, Angiotensin-2-Rezeptor-AB, PAR-1-AB

Inflammatory Markers

CRP, IL1-RA, IL-6, IL1-ß, TNF-a, Neutrophil elastase (NE), IL8, IL-18

Iron Metabolism Parameters

Iron, transferrin, ferritin, transferrin saturation

Glucose Metabolism Parameters

Blood glucose in serum, HbA1c, HbA1c (IFCC), mean glucose concentration

Coagulation/Hemostasis Markers

Fibrinogen, D-Dimer, von Willebrand factor
