# Supplementary material for: Multimodal Web-Based Telerehabilitation for Patients With Post–COVID-19 Condition: Protocol for a Randomized Controlled Trial
Source: JMIR Res Protoc. 2025 May 21;14:e65044. doi: 10.2196/65044 (PMC12138299; doi:10.2196/65044)
Supplement: Multimedia Appendix 3 [file resprot_v14i1e65044_app3.pdf]

Multimedia Appendix 3: Reporting on data processing strategies for determining the VO_{2peak} based on Nolte S. et al. (2023)

**Metabolic cart**	Cycling ergometer (ER 900PC, Ergoline GmbH, Bitz, Germany). Spirometry software (BlueCherry, Geratherm Respiratory GmbH, Bad Kissingen, Germany).
**Measurement mode**	Air volume and gases are continuously measured and analyzed using a breath-by-breath method.
**Software State**	R (R Version 4.4.0; R Studio Version 2023.12.1, Inc., Boston, USA).
**Preprocessing State**	The raw spirometric data (without modifications, transformations) was automatically exported as TXT by the BlueCherry software.
**Preprocessing Strategy**	Unmodified raw data was filtered by using digital filter.
**Processing strategy**	To determine VO_{2peak}, the unmodified raw data was filtered by using a low-pass forward-backward Butterworth filter (each filter: 3rd order, 0.04 Hz cut-off) implemented in the spiro package for R version 0.0.4 [2].
**Rationale**	Based on the current evidence, the digital filter strategy together with the 30-second moving average is the most reliable method for determining VO_{2peak} [1].

Legend: VO_{2peak}: highest oxygen uptake rate achieved during the cardiopulmonary exercise test.

1. Nolte S, Rein R, Quittmann OJ. Data processing strategies to determine maximum oxygen uptake: a systematic scoping review and experimental comparison with guidelines for reporting. Sports Med. Dec 21, 2023;53(12):2463-2475. [FREE Full text] [doi: 10.1007/s40279-023-01903-3] [Medline: 37603201]

2. Nolte S. spiro: an R package for analyzing data from cardiopulmonary exercise testing. J Open Source Softw. Jan 2023;8(81):5089. [doi: 10.21105/joss.05089]
