# Supplementary material for: Multimodal Web-Based Telerehabilitation for Patients With Post–COVID-19 Condition: Protocol for a Randomized Controlled Trial
Source: JMIR Res Protoc. 2025 May 21;14:e65044. doi: 10.2196/65044 (PMC12138299; doi:10.2196/65044)
Supplement: Multimedia Appendix 4 [file resprot_v14i1e65044_app4.pdf]

Multimedia Appendix 4: Reporting on data processing strategies for determining the VE/VCO_2 slope [full slope] based on Nolte S. et al. (2023)

**Metabolic cart**	Cycling ergometer (ER 900PC, Ergoline GmbH, Bitz, Germany).Spirometry software (BlueCherry, Geratherm Respiratory GmbH, Bad Kissingen, Germany).
**Measurement mode**	Air volume and gases are continuously measured and analyzed using a breath-by-breath method.
**Software State**	R (R Version 4.4.0; R Studio Version 2023.12.1, Inc., Boston, USA).
**Preprocessing State**	The raw spirometric data (without modifications, transformations) was automatically exported as TXT by the BlueCherry software.
**Preprocessing Strategy**	Unmodified raw data was used for the statistical analysis.
**Processing strategy**	To determine VE/VCO_2 [full slope], the unmodified raw data was filtered by load phase of cardiopulmonary exercise test [from the start of load to maximum exertion].
**Rationale**	Based on the current evidence, the VE/VCO_2 slope [full slope] is calculated using a linear regression analysis of VE and VCO_2 (Pritchard A. et al 2021).

Legend: VE/VCO_2 slope [full slope]: The minute ventilation/carbon dioxide regression quotient.

Nolte S. spiro: An R package for analyzing data from cardiopulmonary exercise testing. JOSS. 2023;8:5089. doi:10.21105/joss.05089.

Pritchard A, Burns P, Correia J, Jamieson P, Moxon P, Purvis J, et al. ARTP statement on cardiopulmonary exercise testing 2021. BMJ Open Respir Res. Nov 15, 2021;8(1):e001121. [doi: 10.1136/bmjresp-2021-001121] [Medline: 34782330]
