# Supplementary material for: Multimodal Web-Based Telerehabilitation for Patients With Post–COVID-19 Condition: Protocol for a Randomized Controlled Trial
Source: JMIR Res Protoc. 2025 May 21;14:e65044. doi: 10.2196/65044 (PMC12138299; doi:10.2196/65044)
Supplement: Multimedia Appendix 5 [file resprot_v14i1e65044_app5.pdf]

Multimedia Appendix 5: Standard method (V-slope method) and auxiliary criteria for determining first and second ventilatory threshold (Kroidl et al. 2015).

Ventilatory threshold 1	Ventilatory threshold 2
Field 5: VCO_2 vs. VO_2 (V-slope method): First significant increase in VCO_2 compared to VO_2	Field 4: VE vs. VCO_2 VO_2 (V-slope method): Significant increase in VE compared to VCO_2
Field 6: VE/VO_2 and VE/VCO_2 vs. time: Increase in VE/VO_2 without simultaneous increase in VE/VCO_2	Field 6: VE/VO_2 and VE/VCO_2 vs. time: Increase in VE/VCO_2
Field 9: $PETO_2$ vs. time: significant increase in $PETO_2$	Field 9: $PETCO_2$ vs. time: decrease in $PETCO_2$
Field 1: VE vs. time: First significant increase in VE	Field 1: VE vs. time: Second significant increase in VE

Legend: The diagrams are numbered according to Wassermann's 9-field diagram and described according to the y-axis vs. x-axis scheme (Kroidl et al. 2015). The ventilation thresholds are determined by two sports scientists (VE and DTO) and one sports physician (PS). The sports scientists alone, on two different days, without seeing or hearing each other's observations, determined the thresholds for each participant and all cardiopulmonary exercise tests. If the discrepancy (time: min:sec) between the observations (occurrence of thresholds) of the two sports scientists for the same participant and the same cardiopulmonary exercise test was less than 15%, the mean of the two observations was used for further analysis. The sports physician was only consulted if the deviation between the thresholds (time of occurrence) previously determined by the sports scientists for the same person and test was more than 15%. After the threshold values are redetermined by the sports physician, all three observations, i.e. the threshold values determined by two sports scientists and the sports physician, are compared with each other. The observation that showed the greatest deviation was excluded from further analysis. The arithmetic mean was then calculated from the remaining two observations.

Kroidl RF, Schwarz S, Lehnigk B, Fritsch J. Kursbuch Spiroergometrie. Stuttgart, Germany. Georg Thieme Verlag; 2015
