# Supplementary material for: Multimodal Web-Based Telerehabilitation for Patients With Post–COVID-19 Condition: Protocol for a Randomized Controlled Trial
Source: JMIR Res Protoc. 2025 May 21;14:e65044. doi: 10.2196/65044 (PMC12138299; doi:10.2196/65044)
Supplement: Multimedia Appendix 6 [file resprot_v14i1e65044_app6.pdf]

Multimedia Appendix 6: Standard Protocol Items Recommendations for Interventional Trials (Chan, Tetzlaff, Altman, Laupacis, Gøtzsche, Krleža-Jerić et al. 2013)

Section/item	Item No	Description
**Administrative information**		
Title	1	Multimodal Web-Based Telerehabilitation for Patients With Post–COVID-19 Condition: Protocol for a Randomized Controlled Trial
Trial registration	2a	https://drks.de/search/de/trial/DRKS00032394. Registered 28 July 2023 — prospectively registered in German Clinical Trials Register (DRKS)
	2b	
Protocol version	3	Registered 28 July 2023, DRKS00032394
Funding	4	German Social Accident Insurance for non-governmental health and social care institutions (ger. Berufsgenossenschaft für Gesundheitsdienst und Wohlfahrtspflege; BGW); Financial support of the study
Roles and responsibilities	5a	Study leader: Perikles Simon[1]<br>Study coordiantors: Aleksandar Tomaskovic[1], Vincent Weber[1], Other contributors: David T. Ochmann[1], Elmo W. I. Neuberger[1], Alexandra Brahmer[1], Nils Haller[1], Barlo Hillen[1], Kira Enders[1], Viktoria Eggert[1], Peter Zeier[2] & Klaus Lieb[3]<br><br>[1]Department of Sports Medicine, Prevention and Rehabilitation, Institute of Sport Science, Johannes Gutenberg-University Mainz (Germany)<br>[2]Department of Clinical Psychology and Neuropsychology, Institute for Psychology, Johannes Gutenberg-University Mainz (Germany)<br>[3]Clinic for Psychiatry and Psychotherapy, University Medical Center, Johannes Gutenberg-University Mainz (Germany)<br>First Author: Aleksandar Tomaskovic<br>Corresponding Author: Perikles Simon
	5b	Prof. Dr. med Albert Nienhaus:<br>Pappelallee 33/35/37<br>22089 Hamburg<br>Germany<br>Tel.: +49 40 / 20207-3241<br>URL: www.bgw-online.de

	5c	The study is a cooperation project between the Department of Sports Medicine, Prevention and Rehabilitation, Institute of Sport Science, Johannes Gutenberg-University Mainz (GER) and the German Social Accident Insurance for non-governmental health and social care institutions (ger. Berufsgenossenschaft für Gesundheitsdienst und Wohlfahrtspflege; BGW). The study receives funding from the BGW. All authors declare no competing interests. The funding body has not peer-reviewed the manuscript. This sponsor was not involved in the study design, the writing of the manuscript, or the decision to submit the manuscript for publication.
	5d	
**Introduction**		
Background and rationale	6a	Patients affected by post COVID-19 condition (PCC) have long-term and health-related consequences after a severe acute respiratory syndrome Coronavirus-2 infection, manifesting as persistent fatigue, dyspnea/hyperventilation, limitations in daily activities, cognitive impairment as well as mental disorders. Consequences exceed individual health, impacting social systems, and the economy. Existing evidence indicates that multimodal Telerehabilitation (TeR) has the potential to improve cardiorespiratory fitness (CRF), alleviate hyperventilation and reduce care costs. Therefore, we have developed a multimodal, personalized and symptom-oriented TeR program that includes weekly teleconsultations, a pacing approach and exercise therapy for PCC patients with severe symptoms and inability to work.
	6b	Explanation for choice of comparators
Objectives	7	The primary objective of this study is to evaluate the effect of TeR on CRF (VO_{2peak} [ml/min/kg]) and hyperventilation (VE/VCO_2 slope [Full-Slope]), while the secondary aim is to gain insights into the clinical and exercise physiology processes associated with PCC.
Trial design	8	The study is designed as single-center prospective parallel group RCT with a waitlist control design.

Methods: Participants, interventions, and outcomes

Study setting	9	This study is being conducted by the Department of Sports Medicine, Prevention and Rehabilitation at Johannes Gutenberg University Mainz in cooperation with the German Social Accident Insurance for non-governmental health and social institutions (ger. Berufsgenossenschaft für Gesundheitsdienst und Wohlfahrtspflege; BGW). All examinations are carried out in the Department of Sports Medicine, Prevention and Rehabilitation in Mainz (Sports medicine outpatient clinic | Sports Medicine (uni-mainz.de). The BGW (www.bgw-online.de) recruits the patients and arranges the examinations. Total study duration is 16 weeks and consists of an 8-week intervention phase and an 8-week follow-up phase with three examination time points: Baseline assessment (T0) before the intervention, primary assessment (T1) after the intervention and follow-up assessment (T2) after the follow-up phase. All participants receive a consent form one week before T0. They will then undergo a comprehensive baseline assessment at T0 (questionnaires, clinical assessment, CPET and muscle strength testing). A summary of the study phases, examination time points and assessments is provided in Table 1. After the baseline assessment, participants are stratified by gender, age, and peak power output (PPO [Watt/kg body weight]) during the CPET and then randomly assigned to either the intervention group (IG) or the control group (CG) by a blinded researcher using a self-written R script.

Eligibility criteria	10	The study population consist of employees in the German healthcare or social services sector who are unable to work full or part-time due to PCC and have been infected with SARS-CoV-2 as part of their work.<br><br>Inclusion criteria:<br>• Age of ≥18 years<br>• Signature of the consent form<br>• Proven SARS-CoV-2 polymerase chain reaction test provided by medical personnel<br>• Post–COVID-19 condition (PCC) diagnosis by the family or company physician (main diagnosis+ International Classification of Diseases diagnosis code U09.9)<br>• Occupational disability (full or part time) due to PCC<br>• Insured by the German social accident insurance provider for nongovernmental health and social care institutions<br>Exclusion criteria:<br>• No internet access and/or unable or unwilling to participate in the study<br>• Lack of capacity to consent or doubts about capacity to consent<br>• Participation in another rehabilitation program based on exercise and respiratory training. This means that individual passive physical therapy (massage, thermotherapy etc.), psychotherapeutic treatments and visits to the doctor are permitted.<br>• Red flags and absolute contraindications for exercise training and physical activity<br>• Participation in another study<br>• Not able to perform performance diagnostics on the cycle ergometer<br>• Other unspecified factors discouraging study participation
Interventions	11a	Following baseline assessment, patients will be randomly allocated to either the intervention group (IG) or the control group (CG). During the intervention phase (8 weeks), all IG participants receive multimodal and symptom-titrated TeR program that includes weekly teleconsultations, a pacing approach and exercise therapy, while the CG receives treatment as usual. After the primary assessment in the follow-up phase (8 weeks), the IG continued their training but without teleconsultation self-directed, while the CG received the same intervention as the IG followed by follow-up assessment.

11b Based on the baseline assessment results and the algorithm listed in Table, IG participants are assigned to one of three training groups (TG1-3). This enables a personalized and symptom-specific intervention.

Grope	VO_{2peak} (ml/min/kg)	PPO (Watt/kg body weight)	VE/VCO_2 slope (Full-Slope)
TG1	<16	< 1	> 45
TG2	16-20	1 – 1.5	30-45
TG3	> 20	> 1.5	< 30

11c The individual progression or regression of the training load (number of sets, intensity, etc.) depends on the patient's exercise tolerance and can be carried out in consultation with the personal sports therapist

11d Participation in another rehabilitation program based on exercise training is exclusion criteria. At the same time, individual passive physical therapies (massage, thermotherapy, etc.) are permitted.

Outcomes	12	The primary objective of the study is to investigate the effects of the intervention on CRF and hyperventilation. Evaluation of changes from baseline to primary assessment with respect to IG and CG, i.e.:<br>1) Change in CRF (VO_{2peak} [ml/min/kg])[1]<br>2) Change in hyperventilation (VE/VCO_2 slope [Full-Slope])[2]<br><br>[1]CRF: Cardiorespiratory fitness<br>[2]VE/VCO_2 slope: Slope of Minute Ventilation/Carbon Dioxide Production during the complete exercise phase<br><br>The secondary objective is to investigate the effects on other exercise physiological, clinical, and psychosocial variables. Evaluation of changes from baseline to primary assessment with respect to IG and CG, i.e.:<br>1) Changes in power output:<br>a. At maximal exertion (PPO [Watt/kg body weight])<br>b. At the first and second ventilatory threshold (PO [Watt/kg body weight])<br>2) Changes in oxygen uptake efficiency and ventilatory efficiency:<br>a) Oxygen uptake efficiency slope (OUES [VO_2/VElog])<br>b) Oxygen pulse (O_2 pulse [ml/beat])<br>c) Oxygen cost of work ($\Delta VO_2/\Delta WR$ [ml/min/Watt]<br>d) Cardiorespiratory optimal point (COP [VE/VO_2min])<br>e) Ventilatory equivalent for carbon dioxide (ECO_2) and for oxygen (EO_2)<br>f) End-tidal carbon dioxide ($PETCO_2$ [mmHg]) and end-tidal oxygen ($PETO_2$ [mmHg]) concentrations<br>g) Nijmegen Score (NQ Score)<br>3) Change in maximal hand grip strength (HGSmax [kg]) and hand grip strength decline (HGSdiff [kg])<br>4) Changes in blood markers: venous circulating cell-free DNA (cfDNA [ng/ml]) and capillary lactate (La [mmol/l]) concentration<br>5) Adherence to the intervention protocol (% of the prescribed intervention days)<br>6) Changes in clinical condition and functional disability:<br>a. Frequency of occurrence and severity of post exertional malaise (DSQ-SF-PEM [score])<br>b. Canadian Consensus Criteria for diagnosis of myalgic encephalitis/ chronic fatigue syndrome (CCC [Index]) and Bell CFIDS disability scale (Bell [score])<br>7) Changes in the selected psychosocial variables:<br>a) somatization, depression and anxiety (BSI-18 [index])<br>b) severity of insomnia (ISI [index])<br>c) social support (F-SozU K-6 [score])<br>d) optimism and pessimism (SOP2 [score])<br>e) self-efficacy (GSE [score])

Participant timeline	13	Study flowchart: The study consists of three examination time points (T0-T2) and is divided into an 8-week intervention phase and an 8-week follow-up phase. Telerehabilitation: Weekly therapeutic video calls, web-based exercise and respiratory therapy; Training Group Allocation: Allocation of the patients to three training groups based on their baseline CRF and ventilatory efficiency. POTS, respiratory, endurance, and strength: Therapy modalities; Self-directed therapy: The patient carries out the therapy measures independently and without instruction. Pacing: Pacing approach for self-management of the clinical condition and daily activities.

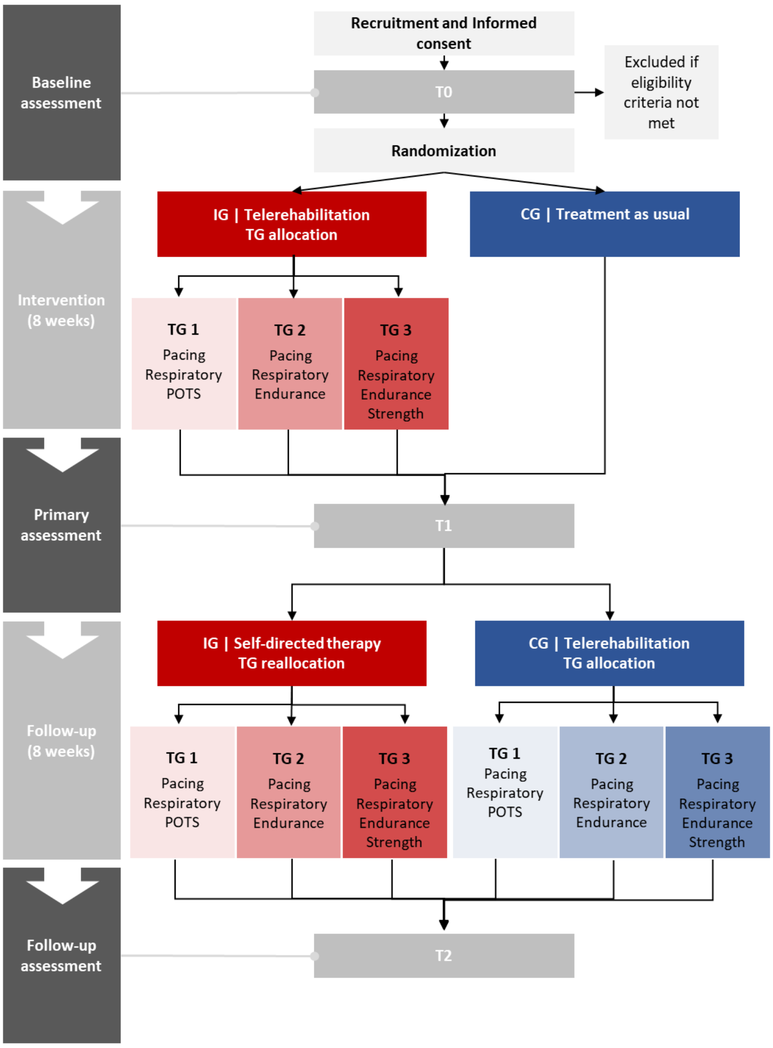

Sample size	14	The sample size estimation is based on the a priori power analysis (repeated measures, interaction between the two) performed with G*Power (Version 3.1.9.7, Düsseldorf, Germany). Assuming a power of 0.8 and a medium to large effect (Cohen's f(V) = 0.35) on VO_{2peak} (in accordance with [1]), 67 participants are required. Taking into account the calculated required sample size and a drop-out rate of 15-20% [2,3], the target number of study participants is set at a total of 80 (devided in40 participants per group).<br><br>[1]Bacon AP, Carter RE, Ogle EA, Joyner MJ. VO2max trainability and high intensity interval training in humans: a meta-analysis. PLoS One. 2013;8:e73182. doi:10.1371/journal.pone.0073182.<br>[2]Pfirrmann D, Haller N, Huber Y, Jung P, Lieb K, Gockel I, et al. Applicability of a Web-Based, Individualized Exercise Intervention in Patients With Liver Disease, Cystic Fibrosis, Esophageal Cancer, and Psychiatric Disorders: Process Evaluation of 4 Ongoing Clinical Trials. JMIR Res Protoc. 2018;7:e106. doi:10.2196/resprot.8607.<br>[3]Haller N, Lorenz S, Pfirrmann D, Koch C, Lieb K, Dettweiler U, et al. Individualized Web-Based Exercise for the Treatment of Depression: Randomized Controlled Trial. JMIR Ment Health. 2018;5:e10698. doi:10.2196/10698.
Recruitment	15	Recruitment is carried out by the Department of Sports Medicine, Prevention and Rehabilitation Mainz in cooperation with the BGW. No active recruitment process, the study participants are provided by the BGW.
**Methods: Assignment of interventions (for controlled trials)**		
Allocation:		Stratified randomization
Sequence generation	16a	After the baseline assessment, participants are stratified by gender, age, and peak power output (PPO [Watt/kg body weight]) during the CPET and then randomly assigned to either the intervention group or the control group by a blinded researcher (Aleksandar Tomaskovic) using a self-written R script (see supplemental material “StratifiedRandomization”).
Allocation concealment mechanism	16b	-
Implementation	16c	The results of the randomization (IG or CG) will be communicated to all participants within a few days after T0 by a blinded researcher (AT) via E-Mail.

Blinding (masking)	17a	After allocation to the intervention, the medical staff/outcome assessors (study physician: Ella Lachtermann and medical assistants) and the study coordinators/data analysts Aleksandar Tomaskovic and Vincent Weber will be blinded.
	17b	In individual cases, e.g. serious adverse events or major technical problems, either the study physician or the study coordinators may be exempted from blinding.

Methods: Data collection, management, and analysis

Data collection methods	18a	Validated questionnaires are completed at each examination time point before the clinical assessment and CPET record various disease- and health-related aspects (s. outcomes). All participants undergo a clinical assessment at all three examination time points prior to CPET. To evaluate changes in CRF and other exercise physiological variables, spiroergometry is performed on ER 900PC cycling ergometer (Ergoline GmbH, Bitz, Germany) using the BlueCherry software (Geratherm Respiratory GmbH, Bad Kissingen, Germany). The exercise test protocol is designed to consist of 4-6 stages, each lasting 2 min, whereby the exercise intensity (in Watt) is continuously increased at the end of each stage. Two test protocols with different intensity increments (10/20 watt) are used, which are tailored to the patient's functional capacity and ensure a test duration of 8-12 minutes, thus maximizing the reliability of the VO_{2peak} determination. To provide accurate information on the data processing strategy to determine the VO_{2peak}, we follow the current Guidelines for Reporting of Data Processing Strategies to Determine Maximum Oxygen Uptake [1].<br><br>[1]Nolte, S., Rein, R., & Quittmann, O. J. (2023). Data Processing Strategies to Determine Maximum Oxygen Uptake: A Systematic Scoping Review and Experimental Comparison with Guidelines for Reporting. Sports Medicine, 53(12), 2463–2475. https://doi.org/10.1007/s40279-023-01903-3
	18b	Standard statistical procedures will be performed according to the intention-to-treat principle.
Data management	19	For data storage, transformation and analysis, Microsoft Excel (Version 2401, Microsoft Corporation, Redmond, WA) and R (R Version 4.4.0; R Studio Version 2023.12.1, Inc., Boston, USA) are used. The data analysts (VW and AT) are blinded and do not know which participant are in the IG and which are in the CG. They also do not know which patient receives which intervention (TG1, TG2 and TG3).

Statistical methods	20a	Descriptive statistics are used for all outcome variables at each test time point to obtain an overview of the changes in all relevant data. Descriptive statistics will include absolute and relative frequencies for categorically scaled data and mean and standard deviation (SD) or median and interquartile range (IQR) for continuously scaled data. The normal distribution is tested with the Shapiro-Wilk test and visual inspection of the data. The homogeneity of variance is tested with the Levene´s test. In case of non-normal distribution, data are log-transformed before statistical analysis to assess normality and variance homogeneity or more robust statistical models like mixed-effects-models are used instead. A repeated-measure analysis of variance, or a linear mixed effects model, will be used to determine the differences between all groups and time points. Statistical significance will be set at alpha < 0.05. The magnitude of the changes between time points as well as the magnitude of differences in changes between the groups will be assessed using effect sizes (ES). Threshold values for ES are 0.2 (small), 0.6 (moderate), 1.2 (large), and 2.0 (very large) [67, 68]. In addition, 95% confidence intervals for the between-groups differences in changes are estimated, and magnitude-based inferences are made with reference to a smallest worthwhile change, which is calculated as 0.2 multiplied by the between-subject variation at T0. The correlation between variables will be assessed using parametric (Pearson) and non-parametric (Spearman Rank) tests, depending on the distribution of the variables. In the case of multiple comparisons, p-values are adjusted using appropriate procedures.
	20b	A repeated measures analysis of variance or a linear mixed effects model will be used to determine the differences not only between the intervention and control groups, but also between the three intervention groups (TG1, 2 and 3).
	20c	Participants who drop out of the intervention protocol or complete less than 60% (i.e. for TG1 less than 17 of pre-defined 24 training sessions) of the prescribed training are defined as dropouts. Adherence to the training requirements will be assessed based on the information provided by the participants via clinical condition and training load diary Participants will continue to be monitored after their withdrawal from the study if they agree.

Methods: Monitoring

Data monitoring	21a	The study plan, documentation, approvals and all other study-related data are stored in a study document folder. All data and documents will be made available to the competent authorities on request. All study-related health data is collected pseudonymously, stored on data carriers and analyzed by the client of the study. The data will only be passed on to third parties, including publication, in anonymized form, i.e. it cannot be assigned to the study participant. Consent to participate in the study can be withdrawn at any time and data can be ordered to be deleted without any disadvantages for the study participants. Any information that becomes available during the course of the study and may be relevant to the study participants will be made known to them by the investigators. Documentation forms and other raw data will be stored for at least 10 years at the Department of Sports Medicine Mainz. At the end of the 10-year retention period, the physical data should be destroyed in a document shredder and all digital data should be deleted.
	21b	The study was funded by the BGW on the basis of a scientific collaboration without commercial interests. This sponsor was not involved in the study design, the preparation of the manuscript or the decision to submit the manuscript for publication. After the initial evaluation, a short interim report on the current status of the study will be prepared and submitted to the sponsor as a criterion for further funding of the study project. Only the principal investigator, Prof. Dr. Perikles Simon, can discontinue the study after approval by the scientific team (Aleksandar Tomaskovic and Vincent Weber).

Harms	22	Study participants can withdraw their consent to participate in the study at any time without giving reasons and without incurring any disadvantages. If they withdraw from the study, it is up to them to decide whether the data already collected and the existing blood samples can continue to be used or should be destroyed. The overall study is terminated in the event of an accumulation of serious adverse events. An adverse event (AE) is an undesirable incident that occurs in a test subject during the course of a study for an examination or therapy. A serious adverse event (SAE) or serious adverse event (SAE) according to § 3 para. 8 GCP-V exists if the AE results in the death of a study participant, is immediately life-threatening, requires an unplanned hospital stay or prolongation of a hospital stay, results in a congenital anomaly or birth defect, or results in a permanent or serious disability or incapacity. In contrast to an adverse reaction, only the temporal relationship is relevant in the case of an adverse event, while a causal relationship is optional (Section 3 para. 6 GCP Regulation GCP-V). A suspected adverse reaction is present if a causal relationship with the study medication is suspected. This is considered unexpected if it does not correspond to the available information on the investigational medicinal product in terms of type or severity.<br><br>The risks listed above due to the physical performance test and venous and capillary blood sampling are considered to be low. There is a chance that the study participants will benefit in terms of their health after the end of the intervention. The low risks involved in the performance diagnostics are explained in detail to the study participants and are written in the declaration of consent. The risks of exercising are minimized by a detailed initial sports medical examination.
Auditing	23	-

Ethics and dissemination

Research ethics approval	24	All procedures have been approved by the Ethics Committee of the Rhineland-Palatinate Medical Association (Mainz, 06/2023; Reg Num: 2023-17082) and are in accordance with the standards of the Declaration of Helsinki.
Protocol amendments	25	Protocol amendments (e.g. inclusion criteria, outcomes) are not planned.

Consent or assent	26a	The written informed consent form will be sent to the study participants by e-mail at least one week before the initial examination (T0). At T0, the study coordinators Aleksandar Tomaskovic and Vincent Weber will provide verbal information and answer any questions about the study that arise after the written and/or verbal information. It explains the nature, procedure and objective of the research project as well as the expected benefits and possible burdens in a generally understandable form
	26b	Additional ethical amendment to recruit another 40 patients (20 PCC patients and 20 control participants) for physiological and biomolecular analyses such as infrared-thrmography, blood analyses during and after the physiological exercise test.
Confidentiality	27	The data of the study participants is recorded using Excel (Microsoft, Redmond, Washington, United States). All personal data is completely pseudonymized immediately after data collection. This means that the data can only be traced back via a key that is only accessible to the study director and the medical director of sports medicine. The data is labeled with the same key as the samples. Confidentiality and data protection are therefore maintained within the scope of this study.
Declaration of interests	28	The study is being carried out in cooperation with the BGW (ger. Berufsgenossenschaft für Gesundheitsdienst und Wohlfahrtspflege) Rhineland-Palatinate. The BGW is named in the publication as the sponsor of this research project. The German Social Accident Insurance expects the results to be made available via OPEN-ACCESS publications. The cooperation is based on scientific, non-commercial interests. The sponsor has not reviewed the manuscript. The sponsor was not involved in the design of the study, the drafting of the manuscript or the decision to submit the manuscript for publication.
Access to data	29	Only the study team, consisting of the study leader Prof. Simon Perikles and the study coordinators Aleksandar Tomaskovic and Vincent Weber, have access to the data. All data and documents will be made available to the relevant authorities (e.g. ethic commsion) on request.
Ancillary and post-trial care	30	This study is not subject to compulsory insurance pursuant to Section 40 para. 1 sentence 3 no. 8 AMG § 40 AMG - Einzelnorm (gesetze-im-internet.de). There is no separate insurance cover for the study participants for the occurrence of damages that are independent of fault. In this respect, claims for damages under the public liability insurance only exist for damages that are attributable to fault and are directly related to patient care.

Dissemination policy	31a	The study results will be published in peer-reviewed journals and presented at symposia, reported objectively irrespective of the outcome.
	31b	PS, AT and VW designed the study. AT, VW, BH and NH planned the intervention modulities and the telerehabilitation. EL performed the clinical examination. VE carried out the telerehabilitation. DTO and PS conducted the weekly consultation with VE. EWIN, AB and KE planned the blood analyses. PZ planned the psychological questionnaire assessments. LB and SP checked the medical plausibility of the study design and evaluations. AT and VW prepared the final manuscript. All authors read and approved the final manuscript.
	31c	The protocol, RCT study and other manuscripts (including data analysis scripts etc.) will be made available via OPEN-ACCESS publications.
**Appendices**		
Informed consent materials	32	A original declaration of consent from the study participants is attached to the documents.
Biological specimens	33	The blood samples are to be stored pseudonymized at -80 °C at the Institute of Sports Science at Johannes Gutenberg University Mainz, Albert-Schweitzer-Str. 22, 55128 Mainz, Germany, for the duration of the study until further processing. Blood samples remaining after completion of the listed analyses will continue to be stored for 10 years after the end of the study. The reason for this is that we are working on the development of a new rapid analysis system for the quantification of cfDNA as an early prognostic marker for on-site surveillance and therapy management of epidemic infections as part of the “EPI-CARE” project funded by the Federal Ministry of Education and Research (link) and these samples would be very valuable for the comparison of measurement methods. After 10 years, the samples will be disposed of properly. The data and/or biomaterials will only be passed on to third parties in anonymized form, i.e. they cannot be assigned to the study participant.

*It is strongly recommended that this checklist be read in conjunction with the SPIRIT 2013 Explanation & Elaboration for important clarification on the items. Amendments to the protocol should be tracked and dated. The SPIRIT checklist is copyrighted by the SPIRIT Group under the Creative Commons “Attribution-NonCommercial-NoDerivs 3.0 Unported” license.

Chan AW, Tetzlaff JM, Altman DG, Laupacis A, Gøtzsche PC, Krleža-Jerić K, et al. SPIRIT 2013 statement: defining standard protocol items for clinical trials. Ann Intern Med. Feb 05, 2013;158(3):200-207. [FREE Full text] [doi:10.7326/0003-4819-158-3-201302050-00583] [Medline: 23295957]
